# Diversity among *bla*_KPC_-containing plasmids in *Escherichia coli* and other bacterial species isolated from the same patients

**DOI:** 10.1038/s41598-018-28085-7

**Published:** 2018-07-06

**Authors:** Tracy H. Hazen, Roberta Mettus, Christi L. McElheny, Sarah L. Bowler, Sushma Nagaraj, Yohei Doi, David A. Rasko

**Affiliations:** 1 Institute for Genome Sciences, University of Maryland School of Medicine, Baltimore, MD 21201 USA; 2 Department of Microbiology and Immunology, University of Maryland School of Medicine, Baltimore, MD 21201 USA; 3 Division of Infectious Diseases and Center for Innovative Antimicrobial Therapy, University of Pittsburgh School of Medicine, Pittsburgh, PA USA; 4 Department of Microbiology, Fujita Health University, Aichi, Japan

## Abstract

Carbapenem resistant *Enterobacteriaceae* are a significant public health concern, and genes encoding the *Klebsiella pneumoniae* carbapenemase (KPC) have contributed to the global spread of carbapenem resistance. In the current study, we used whole-genome sequencing to investigate the diversity of *bla*_KPC_-containing plasmids and antimicrobial resistance mechanisms among 26 *bla*_KPC_-containing *Escherichia coli*, and 13 *bla*_KPC_-containing *Enterobacter asburiae, Enterobacter hormaechei*, *K. pneumoniae, Klebsiella variicola*, *Klebsiella michiganensis*, and *Serratia marcescens* strains, which were isolated from the same patients as the *bla*_KPC_-containing *E. coli*. A *bla*_KPC_-containing IncN and/or IncFII_K_ plasmid was identified in 77% (30/39) of the *E. coli* and other bacterial species analyzed. Complete genome sequencing and comparative analysis of a *bla*_KPC_-containing IncN plasmid from one of the *E. coli* strains demonstrated that this plasmid is present in the *K. pneumoniae* and *S. marcescens* strains from this patient, and is conserved among 13 of the *E. coli* and other bacterial species analyzed. Interestingly, while both IncFII_K_ and IncN plasmids were prevalent among the strains analyzed, the IncN plasmids were more often identified in multiple bacterial species from the same patients, demonstrating a contribution of this IncN plasmid to the inter-genera dissemination of the *bla*_KPC_ genes between the *E. coli* and other bacterial species analyzed.

## Introduction

Carbapenem-resistant *Enterobacteriaceae* (CRE) are a serious public health concern as they are often multidrug-resistant, thus making them exceedingly difficult to treat^1–11^. Also, carbapenem-resistant organisms have been associated with an increased likelihood of death compared with susceptible organisms and have been linked to deadly outbreaks^1^. The carbapenem antibiotics are a primary treatment approach for infections caused by extended-spectrum β-lactamases (ESBL)-producing bacteria, which are resistant to clinically important antibiotics including cephalosporins^2,12^. Thus, the global spread of ESBLs such as CTX-M, has made the increased prevalence of carbapenem resistance an even greater public health concern^2,12,13^.

Carbapenem resistance can result from multiple mechanisms including the activity of carbapenemases, contributions of mutations to β-lactamase function, and changes in outer membrane permeability^14,15^. A recent study using whole genome sequencing demonstrated there was considerable genomic diversity and numerous carbapenem resistance mechanisms among carbapenem-resistant organisms isolated from different healthcare facilities^16^.

Among the most prevalent mechanisms of carbapenem resistance is the *Klebsiella pneumoniae* carbapenemase (KPC), which is one of the class A β-lactamases that confer resistance to carbapenems, penicillins, and cephalosporins^2,14^. Following the initial description of KPC from a *K. pneumoniae* strain identified in 1996^17^, KPCs have been identified in diverse bacteria including *Enterobacter* spp.^18,19^, *Escherichia coli*^20–24^, *Salmonella* spp.^25^, *Acinetobacter* spp.^26^ and *Pseudomonas* spp.^27,28^. The *bla*_KPC_ gene is most often located on the Tn4401 transposon^29–31^; however, *bla*_KPC_ genes have also been identified on other non-Tn4401 mobile elements including Tn1721^23,32,33^. The transposons carrying the *bla*_KPC_ gene have been identified on plasmids with a wide array of incompatibility (Inc) types including IncFII_K_, IncFIA, IncN, IncP, ColE1, and IncA/C, and many of these plasmids have been detected in *bla*_KPC_-containing *E. coli*^22–24,33–36^.

In the current study, we used whole genome sequencing to characterize the diversity of all *bla*_KPC_-containing *E. coli* and any other *bla*_KPC_-containing bacterial species isolated from the same patients in a health system in Pennsylvania over time. This approach allowed us to investigate the diversity of mobile elements and antibiotic resistance genes carried by the *bla*_KPC_-containing *E. coli* among these patients, and also characterize the intergenera transmission of *bla*_KPC_ genes among the *E. coli* and other co-occurring bacterial species. Additionally, long-read sequencing was used to generate a complete genome assembly of a diverse *bla*_KPC_-containing *E. coli* strain, and examine the distribution of the 70-kb *bla*_KPC-3_-containing IncN multidrug resistance plasmid and two additional antibiotic resistance plasmids from this *E. coli* strain.

## Results

### Characteristics of the *bla*_KPC_-harboring *E. coli* and other bacterial species

The 26 *E. coli* strains analyzed in this study were obtained from 26 different patients that received treatment at one of three hospitals in a large health system in Pennsylvania, United States (Table 1). The *E. coli* strains were isolated from at least seven different types of samples including Jackson-Pratt drainage (surgical site), urine, sputum, blood, abdominal drainage/abdominal fistula, tracheal aspirate, and bronchoalveolar lavage (BAL) (Table 1). These *E. coli* strains were selected for genome sequencing because they were initially identified as carbapenem-resistant in the clinical microbiology laboratories, and were later determined to be PCR-positive for a *bla*_KPC_ gene^21,22,37^. The *bla*_KPC_-containing plasmid of *E. coli* strain YD626 was previously characterized by sequencing^34^; however, neither the whole genome of YD626 or any of the other *E. coli* strains included in this study have been previously analyzed using whole genome sequencing. Thus these results are meant to further examine the genomic and plasmid diversity of the *bla*_KPC_-containing *E. coli*. In addition to the *E. coli* strains, 10 of 26 patients had one or more non-*E. coli* cultures that were PCR-positive for a *bla*_KPC_ gene (Table 1). The other bacterial species analyzed were identified as *E. asburiae, E. hormaechei*, *K. pneumoniae, K. variicola*, *K. michiganensis, S. marcescens*, or *P. stuartii*, and were cultured from the same or a subsequent sample from the patients that had the *bla*_KPC_-containing *E. coli* strains (Tables 1 and 2).

**Table 1:** Features of patients and bacteria analyzed in this study.

Patient^a^	Date^b^	Sample^c^	*bla*_KPC_-containing *E. coli* strain	*bla*_KPC_ gene	Other *bla*_KPC_-containing species from the patient^e^
1	2008	JP drainage	YDC107	*bla*_KPC-3_	*K. pneumoniae* YDC121 (*bla*_KPC-3_, 19 days after *E. coli* sample, abdominal abscess), *S. marcescens* YDC107-2 (*bla*_KPC-3_, 2009, sputum)
2	2008	urine	YDC134	*bla*_KPC-3_	
3	2009	urine	YDC304	*bla*_KPC-2_	
4	2009	abdominal drainage	YDC337	*bla*_KPC-3_	*K. michiganensis* YD358 (*bla*_KPC-3_, same day as *E. coli* sample, abdominal fistula), *E. asburiae* YDC337-2 (*bla*_KPC-3_, same day as *E. coli* sample, abdominal fistula)
6	2009	urine	YDC345	*bla*_KPC-2_	
7	2009	urine	YDC354	*bla*_KPC-2_	
8	2009	urine	YD439	*bla*_KPC-3_	
9	2010	BAL	YD509	*bla*_KPC-3_	*S. marcescens* YD509-2 (*bla*_KPC-3_, 2 days after *E. coli* sample, ascites)
10	2010	BAL	YDC419	*bla*_KPC-2_	
11	2011	blood	YDC462	*bla*_KPC-3_	
12	2011	JP drainage	YD648	*bla*_KPC-3_	*K. pneumoniae* YD648-2 (*bla*_KPC-2_, same day, blood)
13	2011	BAL	YD626	*bla*_KPC-2_	*K. variicola* YD626-2 (*bla*_KPC-2_, same sample)
14	2011	BAL	YD649	*bla*_KPC-3_	*E. hormaechei* YDC498 (KPC-3, 70 days after *E. coli*, BAL), *E. hormaechei* YDC518 (KPC-3, 107 days after *E. coli*, blood)
15	2011	urine	YD673	*bla*_KPC-2_	*K. pneumoniae* YDC465 (KPC-3, 153 days before *E. coli*, urine)
16	2011	urine	YD705	*bla*_KPC-2_	
17	2012	urine	YD736	*bla*_KPC-2_	
18	2012	urine	YD748	*bla*_KPC-2_	
19	2012	sputum	YD749	*bla*_KPC-3_	
21	2012	blood	YDC593	*bla*_KPC-2_	
22	2013	BAL	YDC595	*bla*_KPC-2_	
23	2014	blood	YD761	*bla*_KPC-3_	
24	2014	urine	YD762	*bla*_KPC-3_	*K. pneumoniae* YD762-3 (KPC-2, 78 days before *E. coli*, sputum), *K. pneumoniae* YD762-2 (KPC-2, 2 days after *E. coli*, tracheal aspirate)
26	2015	blood	YDC717	*bla*_KPC-2_	
27	2015	urine	YDC735	*bla*_KPC-2_	
28	2015	blood	YDC736-1	*bla*_KPC-2_	*K. michiganensis* YDC736-2 (KPC-2, same sample)
29	2015	BAL	YD789	*bla*_KPC-2_	*Providencia stuartii* YD789-2 (No KPC, 18 days before *E. coli*, BAL)

^a^The patient numbers 1-19 correspond with those in previous studies (Sidjabat *et al*.^37^, Kim *et al*.^21^, O’Hara *et al*.^22^).

^b^The year the sample was collected.

^c^Sample abbreviations: JP, Jackson-Pratt drain; BAL, bronchoalveolar lavage.

^d^The *bla*_KPC_ content, sample date, and sample type corresponding to each strain is indicated in parentheses. *Providencia stuartii* YD789-2 was PCR-positive for a *bla*_KPC_ gene, but the *bla*_KPC_ gene was not detected in the genome assembly.

**Table 2:** Characteristics of the PacBio-sequenced genome assembly of *E. coli* strain YDC107.

Contig	Description	Sequence length (bp)	GC%	Plasmid Inc-types^a^	Resistance genes^b^	GenBank accession no.
YDC107	chromosome	5,198,311	50.63	NA	none	CP025707
pYDC107_184	plasmid (complete)	184,098	51.42	IncFIA(AP001918), IncFIB(AP001918), IncFII(AY458016)	*mrx*, *aadA5, tet(D), sul2, aac(3)-IIa, bla*_TEM-1_*, mphA, sul1, aph(6)-Id, tet(A), dfrA14, aph(3*″*)-Ib, ermB*	CP025708
pYDC107_85	plasmid (incomplete)	85,535	49.68	IncI1	*bla*_CMY-44_	CP025709
pYDC107_70	plasmid (complete)	70,372	53.34	IncN	*bla*_KPC-3_, *bla*_OXA-9_, *bla*_TEM-1_, *sul2, aadA, aac(6*′*)-Ib, aph(3*″*)-Ib, aph(6)-Id, dfrA14*	CP025710
pYDC107_41	plasmid (complete)	41,544	45.34	IncP-1-like *trfA*	none	CP025711
YDC107_phage1	phage	46,701	49.89	NA	none	CP025712
YDC107_phage2	phage	18,617	47.27	NA	none	CP025713

^a^The incompatability type (Inc-type) of each plasmid. NA indicates the inc-typing was not applicable for the chromosome and phage contigs. pYDC107_41 was not predicted to have an inc-type using PlasmidFinder; however, the annotation contained an IncP-1-like *trfA* replication gene.

^b^The antibiotic resistance genes identified on each plasmid. Efflux pumps or mutation-associated resistance genes were also identified on the chromosome that are not shown.

Antimicrobial susceptibility testing demonstrated that the *bla*_KPC_-containing *E. coli* and other bacterial species analyzed were non-susceptible (intermediate or resistant) to between four and 17 of the 21 antimicrobials examined (Supplementary Table S2). All of the *bla*_KPC_-containing *E. coli* and other bacterial species analyzed were resistant to aztreonam (ATM), and all but one of the strains exhibited resistance to ticarcillin-clavulanic acid (TIM) and cefotaxime (CTX) (Supplementary Table S2). All of the *bla*_KPC_-containing *E. coli* strains were susceptible to amikacin (AMK), tigecycline (TGC), colistin (CST), and polymyxin B (PMB) (Supplementary Table S2). In all but one example, the *bla*_KPC_-containing *E. coli* strains exhibited resistance to fewer antibiotics than the other bacterial species isolated from the same patient (Supplementary Table S2). On average, the *bla*_KPC_-containing *E. coli* exhibited resistance to nine antibiotics (range 4 to 14), while the other *bla*_KPC_-containing bacterial species isolated from the same patients had resistance to an average of 13 antibiotics (range 7 to 17) (Supplementary Table S2).

### Genome characteristics of the *E. coli* and other bacterial species analyzed

Whole-genome sequencing was used to investigate the diversity of the mobile elements and antibiotic resistance genes carried by the 26 *bla*_KPC_-containing *E. coli* analyzed in this study. Among the *bla*_KPC_-containing *E. coli* in our study, 42% (11/26) had the *bla*_KPC-3_ gene, while 58% (15/26) had the *bla*_KPC-2_ gene (Supplementary Table S1, Supplementary Fig. S1). *In silico* multilocus sequence typing (MLST) demonstrated that the *E. coli* strains analyzed in this study had nine different STs (Supplementary Table S1). Of the 26 total *E. coli* strains analyzed, 65% (17/26) were identified as ST131, two were ST2521, and the remaining seven strains had different STs (Supplementary Table S1). *In silico* serotype prediction of each *E. coli* genome sequence demonstrated that the *E. coli* strains had 10 different serotypes (Supplementary Table S1). Phylogenomic analysis of the *bla*_KPC_-containing *E. coli* analyzed in this study together with all of the publicly-available *bla*_KPC_-containing *E. coli* in GenBank as of March 2017, highlighted the genomic diversity of *bla*_KPC_-containing *E. coli* analyzed in this study, which were isolated from at least seven different types of clinical samples (Supplementary Fig. S1, Supplementary Table S3). The *bla*_KPC_-containing *E. coli* analyzed in this study were identified in phylogroups B1, B2, D, and F (Supplementary Fig. S1). Although 50% (15/30) of the previously sequenced *bla*_KPC_-containing *E. coli* were in the ST648 MLST lineage, only one of the *bla*_KPC_-containing *E. coli* characterized in this study was identified in this lineage, suggesting that lineages of *E. coli* other than ST648 are involved in dissemination of the *bla*_KPC_ genes among the patients analyzed in this study (Supplementary Fig. S1, Supplementary Table S3). More than half (65%, 17/26) of the *bla*_KPC_-containing *E. coli* characterized in this study were in the ST131 lineage (Supplementary Fig. S1, Supplementary Table S1). All of the previously described *E. coli* in the ST131 lineage possessed the *bla*_KPC-3_ gene, whereas 70% (12/17) of the *E. coli* ST131 genomes sequenced in our study contained the *bla*_KPC-2_ gene (Supplementary Fig. S1).

The 14 non-*E. coli* strains analyzed also had genomes sizes and a GC content consistent with other publicly-available genomes of the same species (Supplementary Table S1). *In silico* determination of the MLST STs of each of the *K. pneumoniae* genomes demonstrated that these strains belonged to the MLST lineages ST37, ST258, and ST454 (Supplementary Table S1). *K. pneumoniae* strains from these MLST lineages have previously been described carrying the *bla*_KPC_ gene^38,39^. In particular, *K. pneumoniae* strains belonging to the ST258 lineage are among the most frequently identified KPC-producing strains, and have contributed to the global spread of *bla*_KPC_ genes^13,39,40^.

### Complete genome sequencing of *bla*_KPC-3_-containing *E. coli* strain YDC107

**Figure 1:**
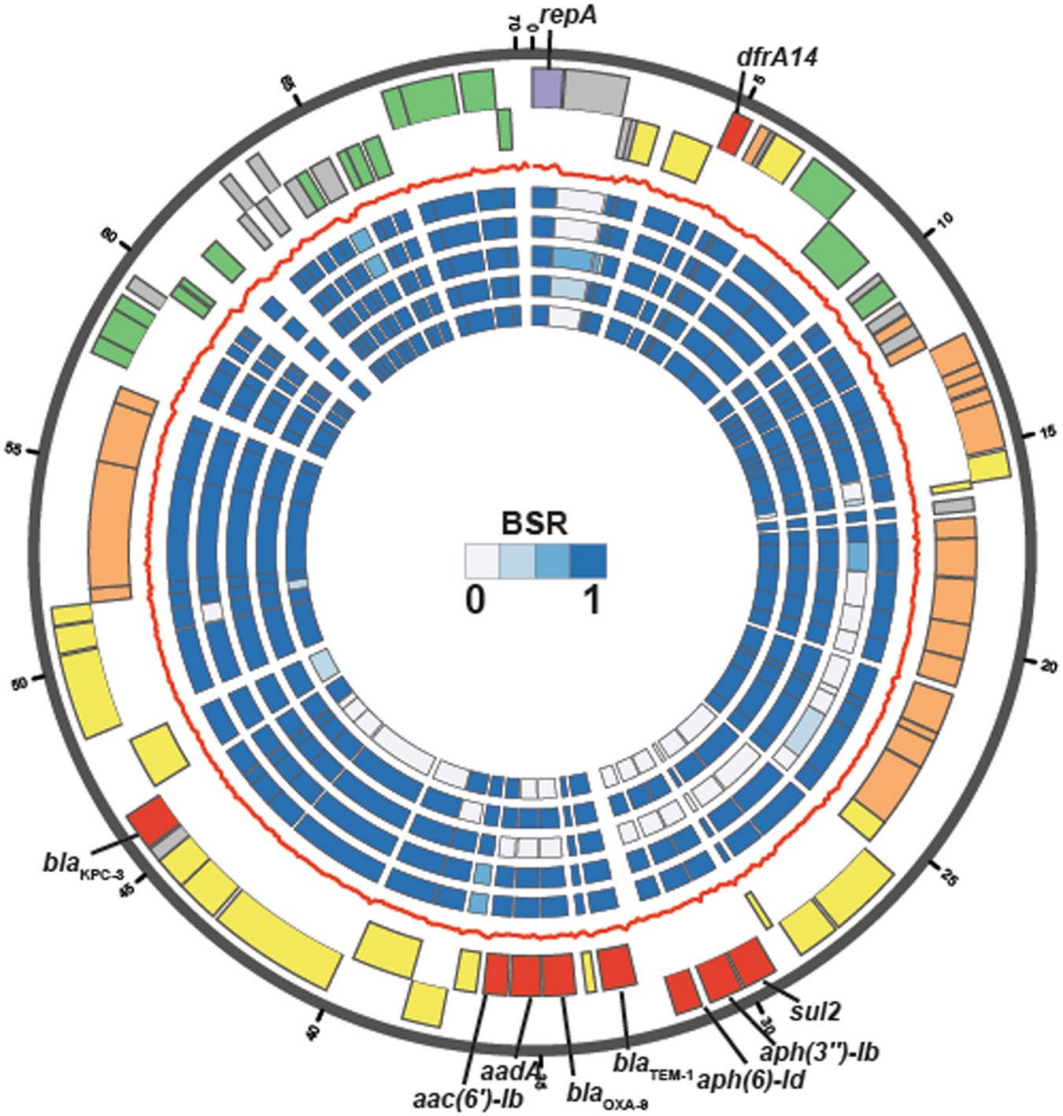
Analysis of the *bla*_KPC-3_-containing plasmid pYDC107_70 from *E. coli* strain YDC107. The two outermost data tracks contain the predicted protein-coding genes on the forward (first track) or reverse (second track) strands of the plasmid. The gene colors indicate their predicted protein functions as follows: antibiotic resistance (red), plasmid replication (purple), plasmid stability (green), conjugative transfer (orange), transposition (yellow), and gray (unknown). The red line separating the gene tracks and the inner heat map tracks is the GC content of the plasmid, calculated on a 100 bp sliding window. The inner heat map tracks (numbered 1 to 5) contain BSR values from the *in silico* detection of each protein-coding gene in the genome sequences of *K. pneumoniae* strain YDC121 (track 1), *S. marcescens* strain YDC107-2 (track 2), both of which were isolated from the same patient as *E. coli* strain YDC107, as well as the previously sequenced *bla*_KPC_-containing IncN plasmids pYD626E (GenBank accession no. KJ933392.1) from *E. coli* strain YD626^34^ (track 3), pBK32602 (GenBank accession no. KU295134.1) from *E. coli* strain BK32602^23^ (track 4), and pECN580 (GenBank accession no. KF914891.1) from *E. coli* strain ECN580^24^ (track 5).

To investigate the plasmid diversity of a *bla*_KPC_-containing *E. coli* strain we generated a complete genome sequence for *E. coli* strain YDC107 (Table 2). *E. coli* strain YDC107 had a predicted serotype of O102:H6, belonged to the MLST lineage ST964, and was present in phylogroup D of the whole-genome phylogeny (Fig. 1). These characteristics make YDC107 a unique strain compared to the majority of the other *bla*_KPC_-containing *E. coli* strains, which primarily belong to the ST131 or ST648 lineages (Supplementary Fig. S1, Supplementary Table S3). The chromosome of *E. coli* strain YDC107 assembled into a single contig that was 5,198,311 bp in length and had a GC content of 50.63% (Table 2). Although the chromosome was a similar size to previously completed *E. coli* genomes it could not be circularized by the assembler, possibly due to the excision of phage regions (Table 2, phage 1 and phage 2) that could be placed both within, and independent from, the chromosome during the assembly process (Table 2). The four additional contigs of the YDC107 genome assembly contained predicted plasmid genes involved in replication, conjugative transfer, and stability (Supplementary Table S4). We have designated the plasmids as follows based on their sequence lengths: pYDC107_184 (184,098 bp), pYDC107_85 (85,535 bp), pYDC107_70 (70,372 bp), and pYDC107_41 (41,544 bp) (Table 2). Three of the plasmids (pYDC107_184, pYDC107_70, and pYDC107_41) circularized during assembly and thus represent complete plasmid sequences. The larger plasmids (pYDC107_184, pYDC107_85, and pYDC107_70) each contained one or more antibiotic resistance genes, whereas the smaller plasmid (pYDC107_41) did not have any predicted antibiotic resistance genes (Table 2, Supplementary Table S7).

### Mobile elements involved in dissemination of the *bla*_KPC_ genes

Whole-genome sequencing also allowed us to investigate the diversity of mobile elements involved in dissemination of the *bla*_KPC_ genes. All but one (*P. stuartii* YD789-2) of the genomes contained either the *bla*_KPC-2_ or *bla*_KPC-3_ gene, and all of the *bla*_KPC_ genes were located on a Tn4401 transposon (Supplementary Table S1). Although the *P. stuartii* strain was initially PCR-positive for a *bla*_KPC_ gene, the *bla*_KPC_ gene was not detected in the genome assembly of this strain, suggesting the strain may have lost the plasmid or *bla*_KPC_ gene prior to sequencing. All of the *bla*_KPC-3_ genes in the genomes sequenced in this study were located on the Tn4401*b* isoform (Supplementary Table S1). The *bla*_KPC-2_ genes were located on the Tn4401*a* isoform in 75% (15/20) of the *bla*_KPC-2_-containing strains analyzed (Supplementary Table S1). The *bla*_KPC-2_ genes in the *E. coli* and other bacterial species from patients 13, 22, and 28 instead carried *bla*_KPC-2_ located on Tn*4401b* (Supplementary Table S1). The genomes of the *E. coli* and non-*E. coli* strains from patients 1, 4, 9, 13, 14, and 28, contained the same *bla*_KPC_ gene and Tn*4401* isoform, while the *E. coli* and non-*E. coli* strains from patients 12, 15, and 24 had different *bla*_KPC_ and Tn*4401* isoform combinations (Supplementary Table S2).

By completing the genome of one of the *bla*_KPC_-containing *E. coli* strains (YDC107) we were able to investigate the similarity of one of the *bla*_KPC_-containing plasmids from an *E. coli* among the other *bla*_KPC_-containing *E. coli* and bacterial species analyzed in this study. The *bla*_KPC-3_ gene of *E. coli* strain YDC107 was identified on a 70-kb IncN plasmid pYDC107_70 along with additional resistance genes including the β-lactamase genes *bla*_OXA-9_ and *bla*_TEM-1_, which typically confer resistance to cephalosporins and aminopenicillins^41,42^ (Fig. 1, Table 2, Supplementary Table S6). The pYDC107_70 plasmid also contained *dfrA14, sul2, aph(3*″*)-Ib, aph(6)-Id*, *aadA*, and *aac(6*′*)-Ib*, which are known to confer resistance to trimethoprim, sulfonamides, and aminoglycosides, respectively (Fig. 1, Table 2, Supplementary Table S6). In addition to the resistance genes, pYDC107_70 carries genes for conjugative transfer and plasmid stability (Supplementary Table S6). Nearly all of the genes of pYDC107_70 were identified in *K. pneumoniae* strain YDC121 and *S. marcescens* strain YDC107-2 also from patient 1, suggesting that this IncN plasmid may have been transferred between these three species (heat map tracks 1 and 2 in Fig. 1). Comparison of the pYDC107_70 plasmid to three previously characterized *bla*_KPC_ plasmids from *E. coli* demonstrated that two of the previously characterized *bla*_KPC_-containing plasmids, pYD626E (GenBank accession number KJ933392.1)^34^ and pECN580 (GenBank accession number KF914891.1)^24^, were missing the *bla*_OXA-9_ gene, and genes that confer resistance to aminoglycosides (*aadA, aac(6*′*)-Ib, aph(6)-Id*, and *aph(3*″*)-Ib*) or sulfonamides (*sul2*) (Fig. 1). In contrast, plasmid pBK32602 (GenBank accession number KU295134.1)^23^ contained nearly all of the protein-coding genes of pYDC107_70, including all of the antibiotic resistance genes (Fig. 1). The *bla*_KPC_ gene of pYDC107 is located on a Tn*4401b* element that appears to be inserted within a Tn*1331-*like element, which is similar to the IncN plasmid pYD626E^34^; however, pYDC107_70 has the *bla*_KPC-3_ gene rather than the *bla*_KPC-2_ gene that was identified on pYD626E (Supplementary Table S6). Plasmids pYDC107_70 and pYD626E also differ among the other resistance genes they carry, with pYD626E carrying a *bla*_LAP-1_, *qnrS1*, and *bla*_TEM-1_ inserted adjacent to the *bla*_KPC_ region, while pYDC107_70 has *bla*_OXA-9_, *sul2, aadA, aac(6*′*)-Ib, aph(6)-Id*, and *aph(3*″*)-Ib* in this same region with *bla*_TEM-1_ (Fig. 1).

**Figure 2:**
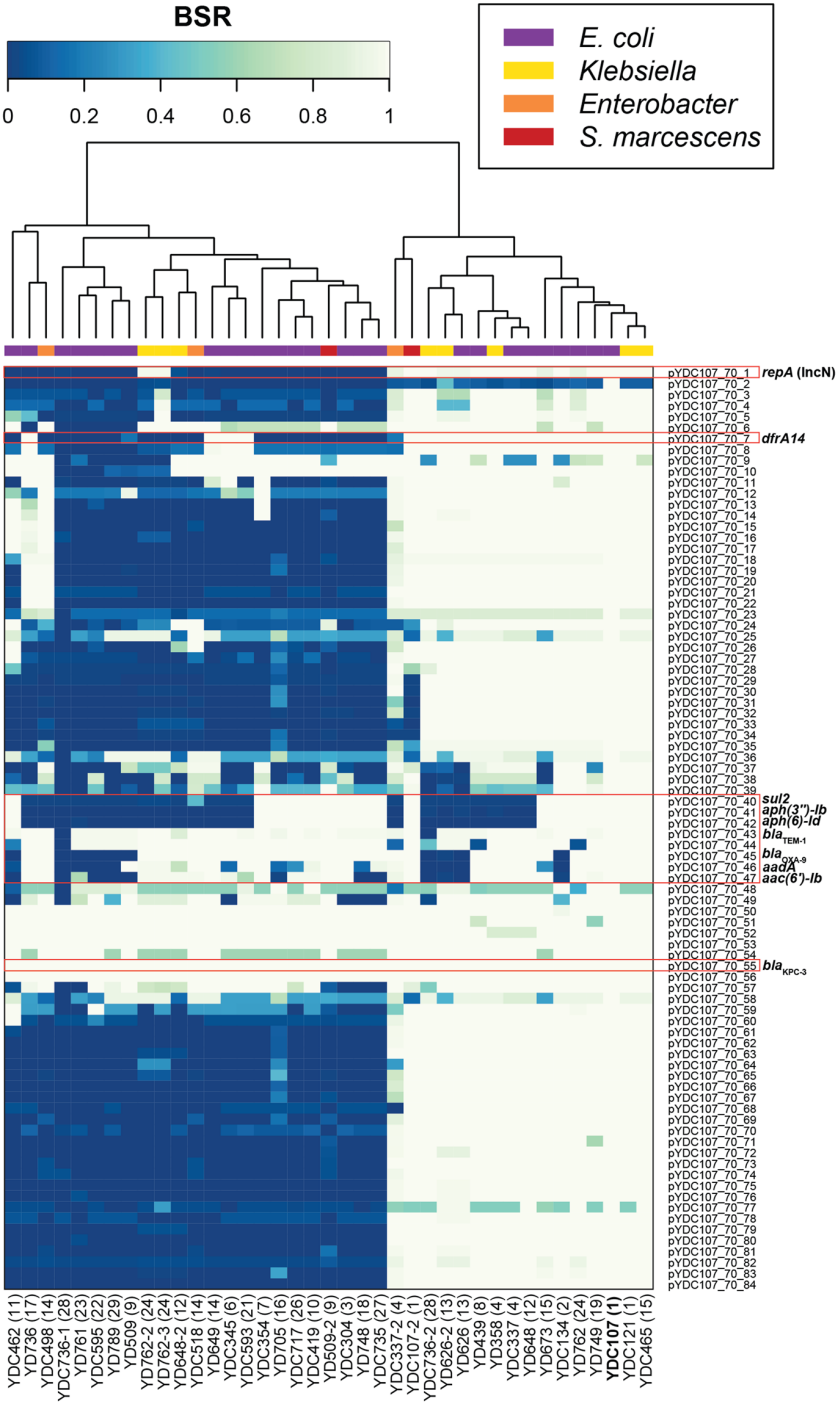
*In silico* detection of plasmid pYDC107_70 in the *bla*_KPC_-containing *E. coli*, and other bacterial species analyzed in this study. The heat map contains BSR values indicating the presence (very light green) or absence (dark blue) of each plasmid gene in each of the genomes. The heat map containing values clustered by column (genomes) was constructed with the heatmap.2 function of gplots using R v.3.4.1. Rows represent each of the protein-coding genes of pYDC107_70, while each column represents a different genome. The label of the *E. coli* YDC107 genome is indicated in bold. The species and/or genus of each genome is indicated by a square at the top of the heat map (see inset legend). The patient number (see Table S1) corresponding to each of the strains is indicated in parentheses next to the strain number.

The *bla*_KPC_-containing contigs ranged in size from 6.5 to 78.8-kb, and 10 of the *bla*_KPC_-containing contigs were identified with markers of the IncFII_K_ plasmids (Supplementary Table S1). The *bla*_KPC_-containing contig of *E. coli* strain YD761 was most similar to an IncFII plasmid (GenBank accession no. CP000670), while the remaining *bla*_KPC_-containing contigs did not contain markers that could be used to identify them to a particular plasmid family (Supplementary Table S1). We further investigated the presence of genes associated with previously characterized *bla*_KPC_-containing plasmids in each of the *E. coli* and other bacterial species analyzed in this study, providing additional information regarding the diversity of potential *bla*_KPC_-containing plasmids in each genome. We used *in silico* analysis to identify genes of the *bla*_KPC-3_-containing IncN plasmid pYDC107_70 described in this study (Fig. 2), and also genes of the previously sequenced IncFII_K_ plasmid pKpQIL (GenBank accession no. GU595196.1) (Supplementary Fig. S4). *In silico* detection of the IncN plasmid pYDC107_70 demonstrated that this plasmid is present with significant similarity in 41% (16/39) of the *bla*_KPC_-containing *E. coli* and other bacterial species analyzed in this study, including nine of the *E. coli* and seven of the other bacterial species (Fig. 2). Detection of the IncFII_K_ plasmid pKpQIL^43^ demonstrated that 12 *E. coli* and one *K. pneumoniae* strain (YD648-2) had genes with significant similarity to this *bla*_KPC_-containing IncFII_K_ plasmid (Supplementary Fig. S4). The other four *K. pneumoniae* genomes analyzed (YDC121, YDC465, YD762-2, and YD762-3) exhibited similarity to genes from several of the regions of the plasmid, but were missing many of the genes of pKpQIL (Supplementary Fig. S4). Overall, 77% (30/39) of the *bla*_KPC_-containing *E. coli* and other bacterial species analyzed in this study have significant similarity to IncN and/or IncFII_K_ plasmids that carry the *bla*_KPC_ gene (Supplementary Table S1, Figs 2 and S4).

### Inter-genera dissemination of the *bla*_KPC_ genes

Comparison of the *bla*_KPC–_containing plasmids among the different species isolated from the same or subsequent samples from the same patients demonstrated a contribution of IncN and IncFII_K_ plasmids to the dissemination of *bla*_KPC_ among the species analyzed in this study. The IncN plasmid pYDC107_70 was present in all of the *E. coli* and other bacterial species isolated from patients 1, 4, 13, and 15 (Fig. 2). While the IncN plasmid was identified in both the *E. coli* and *K. pneumoniae* from patient 15, genes with similarity to those of the IncFII_K_ plasmid were also identified in these strains (Figs 2 and S4, Supplementary Table S1). Interestingly, the *E. coli* strains from patients 12 and 24 had genes with similarity to the IncN plasmid; however, the *K. pneumoniae* strains from each of these patients had genes with similarity to an IncFII_K_ plasmid (Figs 2 and S4, Supplementary Table S1). In contrast, an IncN plasmid was identified in *K. michiganensis* from patient 28, while the *E. coli* from this patient did not carry genes with similarity to the IncN plasmid or to the IncFII_K_ plasmid. The *E. coli* and other species characterized from patients 9 and 14 did not have genes with similarity to those of the IncN plasmid or to the IncFII_K_ plasmid (Supplementary Table S1), suggesting an uncharacterized plasmid may have been involved in the transfer of *bla*_KPC_ among these strains. The *bla*_KPC-3_-containing contigs of *E. coli* strain YD509 and *S. marcescens* strain YD509-2 from patient 9 exhibited 100% nucleotide identity over 85 to 100% of the contig length compared to the 16.9 kb *bla*_KPC-3_-containing plasmid pBK28610 from *E. coli* strain BK28610, which was identified as a novel replicon (GenBank accession no. KU295136.1)^23^.

### Additional antibiotic resistance genes and plasmids carried by the *bla*_KPC_-containing *E. coli* and other bacterial species

The *E. coli* and other bacterial species analyzed contained at least 37 different plasmid types, including members of the IncA/C, IncN, IncI1, IncFII_k_, and IncFIB plasmid families (Supplementary S1), which have been previously described carrying antibiotic resistance genes^44^. Other types of resistance genes identified in the genomes included *qnrS1, aadA, sul1, dfrA14, mphA*, and *cat*, among many others, which confer resistance to fluoroquinolones, aminoglycosides, sulfonamides, trimethoprim, macrolides, and chloramphenicol, respectively (Supplementary Table S2). Comparison of the resistance gene content identified in strains from the same patient demonstrated that the strains contained many, but not all, of the same resistance genes (Supplementary Table S2). For example, among the three strains from Patient 14 (*E. coli* strain YD649, and *E. hormaechei* strains YDC498 and YDC518), all three of the genomes contained *aadA, sul1, qnrA1, aac(6*′*)-Ib, ant(2*″*)-Ia* (Supplementary Table S2). However, *E. coli* strain YD649 also contained a dihydrofolate reductase gene (*dfrA14*), which was absent from the *E. hormaechei* strains, while the *E. hormaechei* strains contained a chloramphenicol acetyltransferase gene (*cat*) that was absent from *E. coli* strain YD649 (Supplementary Table S2). In addition to having similar antibiotic resistance gene content, all three of the strains from Patient 14 also had an IncA/C_2_ plasmid (Supplementary Table S1, Supplementary Table S2), which have been previously characterized as large multidrug resistance plasmids^44^.

Completion genome sequencing of *E. coli* strain YDC107 allowed us to characterize and investigate the distribution of not only the *bla*_KPC_-containing plasmid from this *E. coli* strain, but also any additional plasmids harboring antibiotic resistance genes. *In silico* detection of the largest plasmid, pYDC107_184, among all of the *bla*_KPC_-containing sequenced in this study demonstrated that none of the other genomes analyzed had the entire plasmid; however, 21 of the other *E. coli* genomes had similarity to several regions of this plasmid (Supplementary Fig. S2, group II). The regions that were detected included genes from the antibiotic resistance region, conjugative transfer genes, and genes involved in plasmid stability including partitioning and toxin-antitoxin genes (Supplementary Fig. S2, Supplementary Table S4). The second largest plasmid of *E. coli* strain YDC107 is plasmid pYDC107_85, which is an IncI1 plasmid that has genes for conjugative transfer, but contains only a single antibiotic resistance gene (Supplementary Fig. S3, Supplementary Table S5). Detection of the pYDC107_85 genes in all of the *E. coli* and other bacterial species analyzed in this study demonstrated that many of the genes on this IncI1 plasmid are present in five of the other *E. coli* genomes (Supplementary Fig. S3). Several regions, including the region with the *bla*_CMY-44_ gene were absent from these five *E. coli* genomes; however, additional sequencing would be necessary to complete the IncI1 plasmids from these *E. coli* strains to determine whether the entire plasmid is present among these strains. Finally, detection of the smallest plasmid of *E. coli* strain YDC107, pYDC107_41, demonstrated that this plasmid is not present in any of the other *bla*_KPC_-containing *E. coli* or other bacterial species analyzed in this study.

## Discussion

In the current study we used whole genome sequencing to gain insight into the mobile genetic elements and antibiotic resistance genes carried by *bla*_KPC_-containing *E. coli* and other bacterial species isolated from the same or subsequent patient samples. The *bla*_KPC_-containing *E. coli* strains analyzed in this study were isolated from at least seven different types of clinical samples and included not only members of the ST131 and ST648 lineages, which are well known to carry antibiotic resistance determinants^13,16^, but also included other genomically diverse *E. coli* strains. The majority (77%) of the *bla*_KPC_-containing genomes in the current study had genes with similarity to a *bla*_KPC_-containing IncN and/or IncFII_K_ plasmid. The IncFII_K_ plasmid family includes the pKpQIL-like plasmids, which have been implicated in the spread of *bla*_KPC_ genes^22,35,38,43,45,46^. Also, a previous study demonstrated that IncN and IncFII_K_ plasmids were involved in the inter-genera transfer of *bla*_KPC_ genes between *K. pneumoniae* and other bacterial species including *E. coli, Enterobacter* species, and *Citrobacter* species^47^. While the IncFII_K_ plasmids were prevalent among the *E. coli* analyzed in the current study^22^, the IncN plasmid was more frequently identified in all of the *bla*_KPC_-containing *E. coli* and other species from the same patient, suggesting this plasmid may have had a greater contribution to the inter-genera spread of *bla*_KPC_ among the patients analyzed in this study. Interestingly, the *E. coli* from two patients carried an IncN plasmid while the other bacterial species from the same patients had an IncFII_K_-like plasmid (patients 12 and 24), indicating multiple modes of acquisition of *bla*_KPC_ among the bacterial species in these patients. Further functional studies are necessary to investigate whether the IncN plasmid may be more likely to be transferred between *E. coli* and other co-occurring bacterial species within a patient.

Complete genome sequencing of *E. coli* strain YDC107 allowed us to describe the *bla*_KPC_ plasmid and other co-occurring plasmids in this strain. Interestingly, only the IncN *bla*_KPC_ plasmid, pYDC107_70, from *E. coli* strain YDC107 appears to have been transferred and maintained among the two other *bla*_KPC_-containing bacterial species from this patient. Also, comparison of the *bla*_KPC-3_-containing IncN plasmid pYDC107_70 with the previously sequenced *bla*_KPC-2_-containing IncN plasmid from an *E. coli* that was isolated three years after the isolation of the *E. coli* strain YDC107 demonstrated that while these plasmids have a conserved backbone, they differ in their resistance gene content. This was similar to the comparison of pYDC107_70 among *bla*_KPC_-containing *E. coli* and other bacterial species analyzed in this study, which demonstrated that the plasmid was highly conserved, and most of the sequence variability was detected in the antibiotic resistance gene regions. Thus, the IncN plasmids involved in dissemination of *bla*_KPC_ genes among patients in this health system have likely undergone changes in their resistance gene regions over time.

In summary, our findings demonstrate that *bla*_KPC_-containing IncN and IncFII_K_ plasmids are the most frequently identified plasmids among *bla*_KPC_-containing *E. coli* and other bacterial species from the same patients in a health system in Pennsylvania over 6 years. Whole-genome sequencing demonstrated the each of the *bla*_KPC_-containing *E. coli* and other species analyzed contained numerous antibiotic resistance genes that may be harbored on the same or a different plasmid than the *bla*_KPC_ gene. Also, sequence analyses demonstrated that the *bla*_KPC_-containing IncN plasmid from patients in this health system has undergone modifications over time, which have occurred primarily in the resistance gene regions. Overall, our findings highlight the need for additional studies to investigate whether *E. coli* has an important role as a reservoir of *bla*_KPC_ genes, which may be disseminated to co-occurring species that includes difficult-to-treat or outbreak-associated pathogens such as *K. pneumoniae*. Further studies are also necessary to better understand the dynamic nature of *bla*_KPC_-containing plasmids, and to determine how environmental and host factors can drive changes in resistance gene content and/or inter-genera plasmid dissemination, and whether these changes influence the clinical outcome of the patient.

## Methods

### Bacterial strains and antibiotic susceptibilities

*Escherichia coli* clinical strains that were reported as resistant to ertapenem were collected at the clinical microbiology laboratories at two teaching hospitals in Pittsburgh, PA between 2009 and 2015. Those that tested positive for *bla*_KPC_ by conventional PCR were included in this study. When ertapenem-resistant strain(s) from other species were identified from the same patients from whom *bla*_KPC_-positive *E. coli* strains were identified and tested positive for *bla*_KPC_, these strains were also included. A limited number of the strains included in this study have been reported previously^21,22,37^. The *bla*_KPC_-containing strains analyzed in this study were tested for their susceptibility to 21 antibiotics by determining their minimum inhibitory concentrations (MIC) to each antibiotic using the Sensititre Gram-negative plates GNX2F (Thermo). The designations of susceptible, intermediate, or resistant were assigned based on the CLSI 2017 breakpoints for each species^48^.

### Genome sequencing and assembly

Genomic DNA was extracted using the Sigma GenElute bacterial genomic DNA kit (Sigma-Aldrich; St. Louis, MO). All the genomes were sequenced using paired-end 500 bp insert libraries on the Illumina HiSeq. 4000 and the resulting 150 bp Illumina reads were assembled using SPAdes v.3.7.1^49^. The final assemblies were filtered to contain only contigs that were ≥500 bp in length and had ≥5 × k-mer coverage. *E. coli* strain YDC107 was also sequenced using long-read sequencing to obtain a complete genome assembly, including any possible plasmids as previously described^50^.

### *In silico* multilocus sequence typing, plasmid typing, serotyping, and antibiotic resistance gene detection

The MLST STs of each of the *E. coli* genome assemblies were determined based on the MLST scheme developed by Wirth *et al*.^51^. The sequences of the seven MLST loci (*adk, gyrB, fumC, icd, mdh, purA*, and *recA*) were located in each of the *E. coli* genomes using an in-house perl script. The sequences were queried against the BIGSdb database^52^ to obtain allele numbers, and the allelic profile of each strain was submitted to BIGSdb to obtain the ST for each of *E. coli* genomes analyzed (Supplementary Table S3). The STs of the *K. pneumoniae* genomes were determined by uploading each genome assembly to the BIGSdb whole-genome MLST prediction software on the Institut Pasteur website (http://bigsdb.pasteur.fr).

Plasmids were detected in each of the genome assemblies using PlasmidFinder v.1.3^53^ using the default 95% nucleotide identity threshold. The molecular serotype of each *E. coli* genome was determined using SerotypeFinder v.1.1 (https://cge.cbs.dtu.dk/services/SerotypeFinder/) with the default settings of an 85% nucleotide identity threshold and 60% minimum alignment length^54^. Antibiotic resistance genes were detected in each of the genome assemblies using the resistance gene identifier (RGI) of the comprehensive antibiotic resistance database (CARD) v.1.1.8, with perfect or strict identification criteria^55^. The Tn4401 isoforms were determined by comparing the region surrounding each *bla*_KPC_ gene to the sequences of the previously described Tn4401 isoforms and identifying the presence or absence of deletions that are characteristic of each isoform^56^.

### Phylogenomic analysis

The 26 *E. coli* genomes sequenced in this study were compared with 30 publicly-available *bla*_KPC_-containing *E. coli* genomes as of March 2017, and a collection of 34 diverse *E. coli* and *Shigella* reference genomes using the single nucleotide polymorphism (SNP)-based *In Silico* Genotyper (ISG) as previously described^57,58^. The SNPs were predicted relative to the genome of *E. coli* strain IAI39 (GenBank accession number NC_011750.1) from phylogroup F. ISG identified 225,323 conserved SNP sites that were used to infer a maximum-likelihood phylogeny using RAxML v7.2.8^59^, with the GTR model of nucleotide substitution, the GAMMA model of rate heterogeneity, and 100 bootstrap replicates.

### *In silico* detection of plasmid genes

The predicted protein-coding genes on the *E. coli* strain YDC107 plasmids (pYDC107_184, pYDC107_85, pYDC107_70, and pYDC107_41) were identified in each of the genomes analyzed in this study using large-scale BLAST score ratio (LS-BSR) analysis as previously described^60,61^. The plasmid genes were compared to the *E. coli* genomes using BLASTN^62^.

Clustered heat maps were generated with the BSR values indicating the presence or absence of protein-coding genes of plasmids pYDC107_184, pYDC107_85, pYDC107_70, and the IncFII_K_ plasmid pKpQIL (Genome accession no. GU595196.1) in each of genomes analyzed. The heat maps were generated using the heatmap.2 function of gplots v. 3.0.1 in R v. 3.4.1, and the genomes in each heat map were clustered using the default complete linkage method with Euclidean distance estimation. The plasmid map of pYDC107_70 was generated using Circos 0.69-4^63^. The heat map tracks of the circular plasmid plot contain the BLASTN BSR values of each of the protein-coding genes of pYDC107_70 compared with the plasmids and genomes described in the figure legend.

### Data availability

The genome assemblies generated in this study are deposited in GenBank under the accession numbers listed in Supplementary Table S1.

## Electronic supplementary material

Supplemental Information
