# Supplementary material for: Diversity among blaKPC-containing plasmids in Escherichia coli and other bacterial species isolated from the same patients
Source: Sci Rep. 2018 Jul 6;8:10291. doi: 10.1038/s41598-018-28085-7 (PMC6035167; doi:10.1038/s41598-018-28085-7)
Supplement: Supplementary file 1 — Supplemental Information [file 41598_2018_28085_MOESM1_ESM.pdf]

Supplemental Information for:

Diversity among *bla*$_{KPC}$-containing plasmids in *Escherichia coli* and other bacterial species isolated from the same patients

Tracy H. Hazen[1,2], Roberta Mettus[3], Christi L. McElheny[3], Sarah L. Bowler[3], Sushma Nagaraj[1], Yohei Doi,[3,4*] and David A. Rasko[1,2*]

[1]Institute for Genome Sciences, [2]Department of Microbiology and Immunology, University of Maryland School of Medicine, Baltimore, MD, 21201, [3]Division of Infectious Diseases and Center for Innovative Antimicrobial Therapy, University of Pittsburgh School of Medicine, Pittsburgh, PA, USA, [4] Department of Microbiology, Fujita Health University, Aichi, Japan

Running title: Diversity of *bla*$_{KPC}$-containing plasmids

* Corresponding Author:
David A. Rasko
University of Maryland School of Medicine
Institute for Genome Sciences
Department of Microbiology and Immunology
801 W. Baltimore Street, Suite 619
Baltimore, MD 21201
Phone: 410.706.6774
E-mail: drasko@som.umaryland.edu

Yohei Doi
Division of Infectious Diseases,
Center for Innovative Antimicrobial Therapy,
University of Pittsburgh School of Medicine,
S829 Scaife Hall, 3550 Terrace Street,
Pittsburgh, PA 15261, USA.
Phone: +1-412-648-9445;
E-mail: yod4@pitt.edu

File contains:
4 Supplemental Figures.
7 Supplemental Tables.

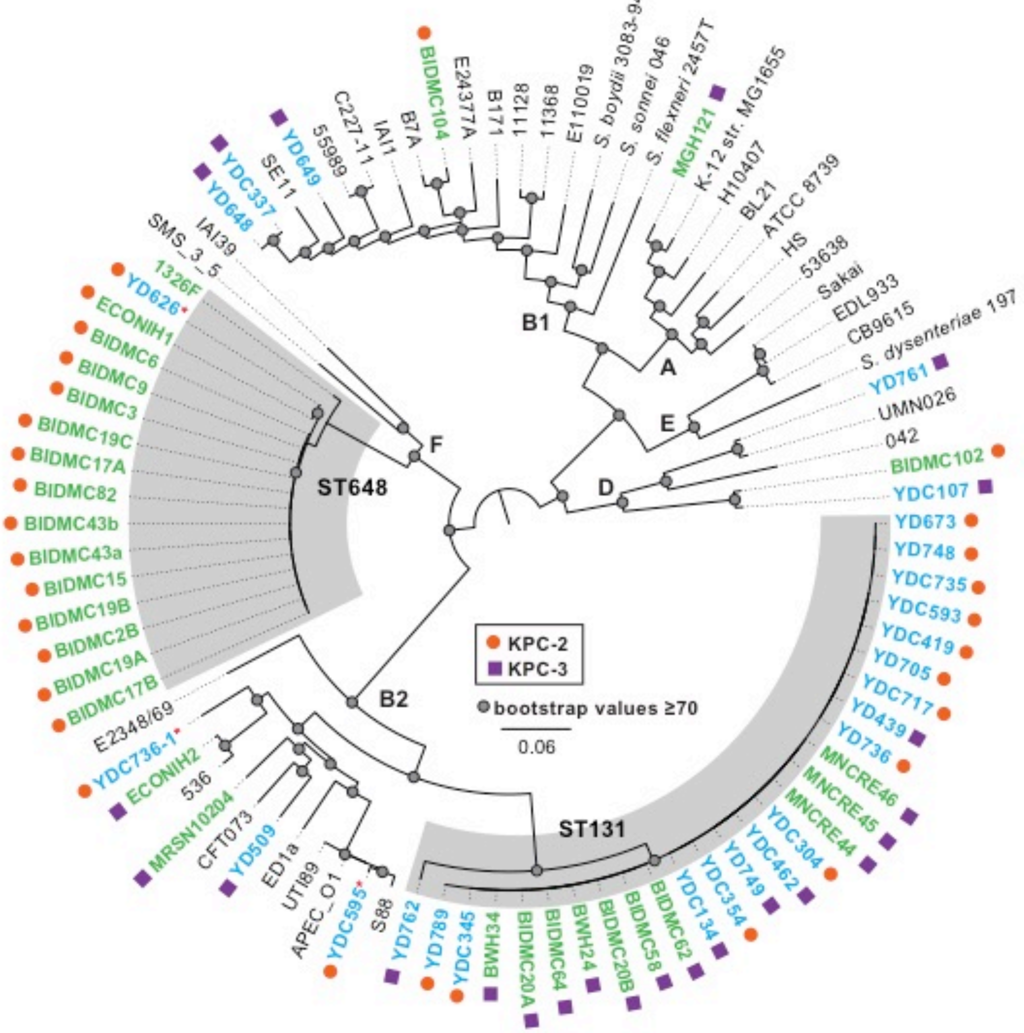

Figure S1. Phylogenomic analysis of the bla_{KPC}-containing *E. coli* analyzed. A SNP-based phylogeny with 225,323 conserved SNP sites relative to the genome of *E. coli* strain IAI39 of phylogroup F was inferred to compare the genomes of bla_{KPC}-containing *E. coli* sequenced in this study with publicly-available bla_{KPC}-containing *E. coli*. The bla_{KPC}-containing *E. coli* genomes analyzed in the current study have blue strain labels, while the previously-sequenced bla_{KPC}-containing *E. coli* that are publicly available have green strain labels. The genomes that contained the bla_{KPC-2} gene are indicated by an

orange circle, while the genomes with the *bla*$_{KPC-3}$ gene are indicated by a purple rectangle. The three genomes that contained *bla*$_{KPC-2}$ on Tn*4401b* are labeled with a red asterisk, while the other *bla*$_{KPC-2}$-containing genomes have *bla*$_{KPC-2}$ on Tn*4401a* and the *bla*$_{KPC-3}$ genomes have *bla*$_{KPC-3}$ on Tn*4401b*. Boldface letters designated the *E. coli* phylogroups (A, B1, B2, D, E, and F). A gray circle indicates bootstrap values ≥70, while the scale bar indicates the approximate distance of 0.06 nucleotide substitutions per site.

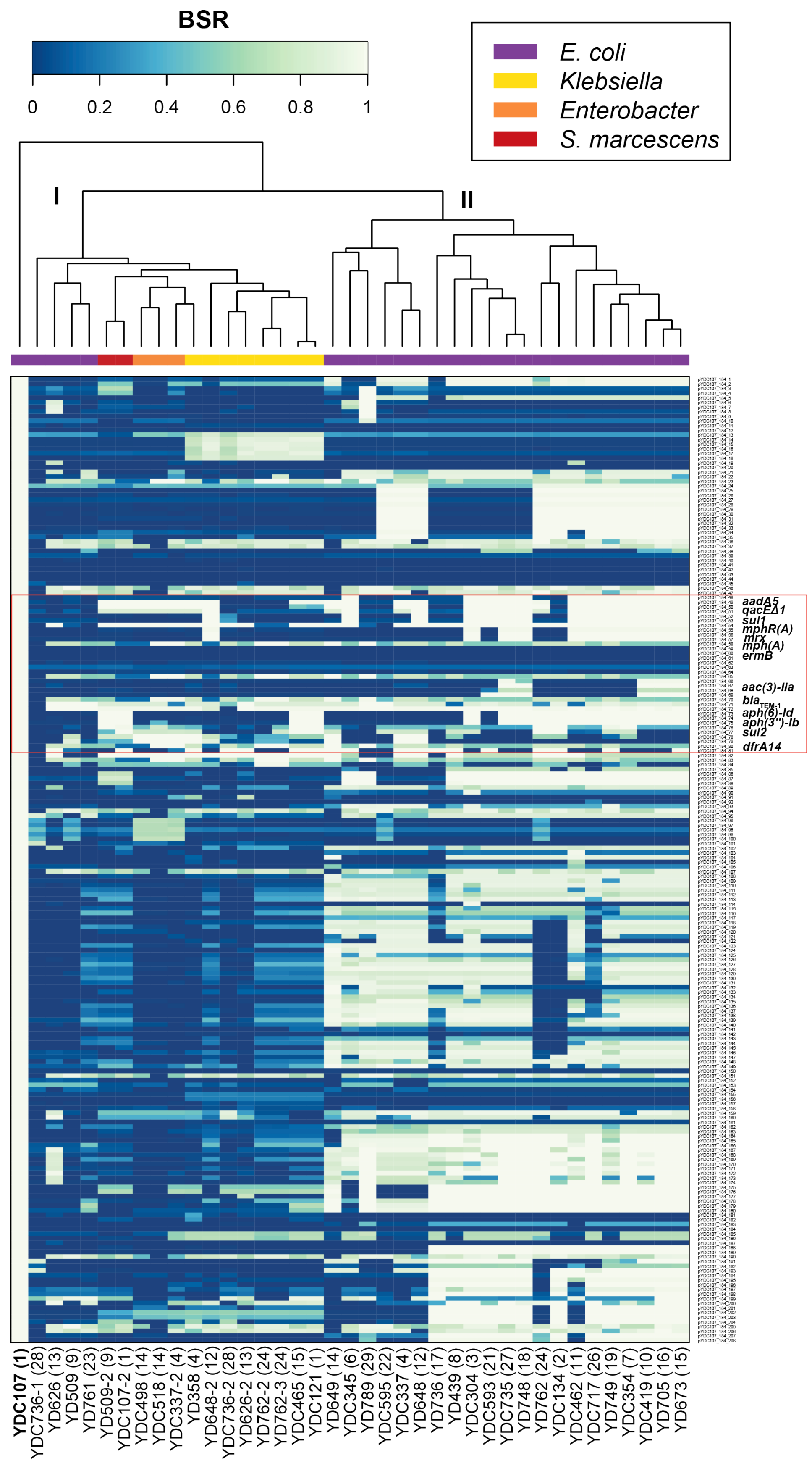

BSR
0
0.2
0.4
0.6
0.8
1
E. coli
Klebsiella
Enterobacter
S. marcescens
I
II
aadA5
qacEΔ1
sul1
mphR(A)
mrx
mph(A)
ermB
aac(3)-IIa
bla TEM-1
aph(6)-Id
aph(3'')-Ib
sul2
dfrA14
YDC107 (1)
YDC736-1 (28)
YD626 (13)
YD509 (9)
YD761 (23)
YD509-2 (9)
YDC107-2 (1)
YDC498 (14)
YDC518 (14)
YDC337-2 (4)
YD358 (4)
YD648-2 (12)
YDC736-2 (28)
YD626-2 (13)
YD762-2 (24)
YD762-3 (24)
YDC465 (15)
YDC121 (1)
YD649 (14)
YDC345 (6)
YD789 (29)
YDC595 (22)
YDC337 (4)
YD648 (12)
YD736 (17)
YD439 (8)
YDC304 (3)
YDC593 (21)
YDC735 (27)
YD748 (18)
YD762 (24)
YDC134 (2)
YDC462 (11)
YDC717 (26)
YD749 (19)
YDC354 (7)
YDC419 (10)
YD705 (16)
YD673 (15)

Figure S2. *In silico* detection of plasmid pYDC107_184 in the bla_{KPC}-containing *E. coli* and other bacterial species isolated from the same patients. The heat map contains BSR values indicating the presence (very light green) or absence (dark blue) of each of the pYDC107_184 protein-coding genes in each of the genomes analyzed in this study. The heat map was constructed with the heatmap.2 function of gplots with R v.3.4.1. Rows represent the protein-coding genes of pYDC107_184, while each column represents a different genome. The species and/or genus of each genome is indicated by a square at the top of the heat map (see Figure legend). The label of the *E. coli* YDC107 genome is in bold. A red box denotes the region of the plasmid that containing resistance genes, which are labeled on the right margin by their gene names. The patient number (see Table S1) corresponding to each of the strains is indicated in parentheses next to the strain number.

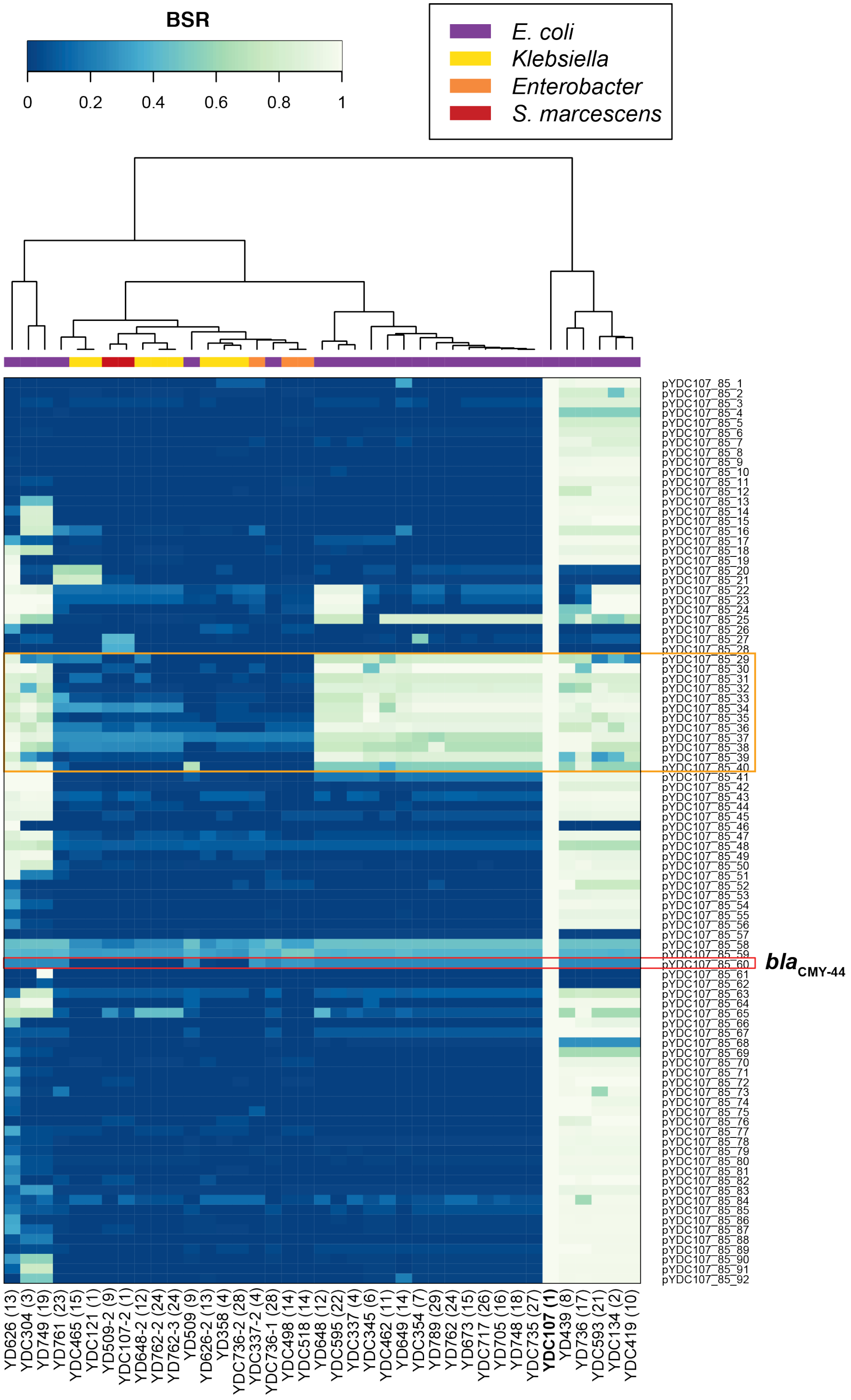

BSR
0
0.2
0.4
0.6
0.8
1
E. coli
Klebsiella
Enterobacter
S. marcescens
pYDC107_85_1
pYDC107_85_2
pYDC107_85_3
pYDC107_85_4
pYDC107_85_5
pYDC107_85_6
pYDC107_85_7
pYDC107_85_8
pYDC107_85_9
pYDC107_85_10
pYDC107_85_11
pYDC107_85_12
pYDC107_85_13
pYDC107_85_14
pYDC107_85_15
pYDC107_85_16
pYDC107_85_17
pYDC107_85_18
pYDC107_85_19
pYDC107_85_20
pYDC107_85_21
pYDC107_85_22
pYDC107_85_23
pYDC107_85_24
pYDC107_85_25
pYDC107_85_26
pYDC107_85_27
pYDC107_85_28
pYDC107_85_29
pYDC107_85_30
pYDC107_85_31
pYDC107_85_32
pYDC107_85_33
pYDC107_85_34
pYDC107_85_35
pYDC107_85_36
pYDC107_85_37
pYDC107_85_38
pYDC107_85_39
pYDC107_85_40
pYDC107_85_41
pYDC107_85_42
pYDC107_85_43
pYDC107_85_44
pYDC107_85_45
pYDC107_85_46
pYDC107_85_47
pYDC107_85_48
pYDC107_85_49
pYDC107_85_50
pYDC107_85_51
pYDC107_85_52
pYDC107_85_53
pYDC107_85_54
pYDC107_85_55
pYDC107_85_56
pYDC107_85_57
pYDC107_85_58
pYDC107_85_59
pYDC107_85_60
pYDC107_85_61
pYDC107_85_62
pYDC107_85_63
pYDC107_85_64
pYDC107_85_65
pYDC107_85_66
pYDC107_85_67
pYDC107_85_68
pYDC107_85_69
pYDC107_85_70
pYDC107_85_71
pYDC107_85_72
pYDC107_85_73
pYDC107_85_74
pYDC107_85_75
pYDC107_85_76
pYDC107_85_77
pYDC107_85_78
pYDC107_85_79
pYDC107_85_80
pYDC107_85_81
pYDC107_85_82
pYDC107_85_83
pYDC107_85_84
pYDC107_85_85
pYDC107_85_86
pYDC107_85_87
pYDC107_85_88
pYDC107_85_89
pYDC107_85_90
pYDC107_85_91
pYDC107_85_92
bla_{CMY-44}
YD626 (13)
YDC304 (3)
YD749 (19)
YD761 (23)
YDC465 (15)
YDC121 (1)
YD509-2 (9)
YDC107-2 (1)
YD648-2 (12)
YD762-2 (24)
YD762-3 (24)
YD509 (9)
YD626-2 (13)
YD358 (4)
YDC736-2 (28)
YDC337-2 (4)
YDC736-1 (28)
YDC498 (14)
YDC518 (14)
YD648 (12)
YDC595 (22)
YDC337 (4)
YDC345 (6)
YDC462 (11)
YD649 (14)
YDC354 (7)
YD789 (29)
YD762 (24)
YD673 (15)
YDC717 (26)
YD705 (16)
YD748 (18)
YDC735 (27)
YDC107 (1)
YD439 (8)
YD736 (17)
YDC593 (21)
YDC134 (2)
YDC419 (10)

Figure S3. *In silico* detection of plasmid pYDC107_85 in the bla_{KPC}-containing *E. coli* and other bacterial species isolated from the same patients. The heat map contains BSR values indicating the presence (very light green) or absence (dark blue) of each plasmid gene in each of the genomes. The heat map was constructed with the heatmap.2 function of gplots using R v.3.4.1. Rows represent each of the protein-coding genes of pYDC107_85, while each column represents a different genome. The label of the *E. coli* YDC107 genome is in bold. The species and/or genus of each genome is indicated by a square at the top of the heat map (see inset legend). A red box indicates the location of the antibiotic resistance gene $bla_{CMY\text{-}44}$. The patient number (see Table S1) corresponding to each of the strains is indicated in parentheses next to the strain number.

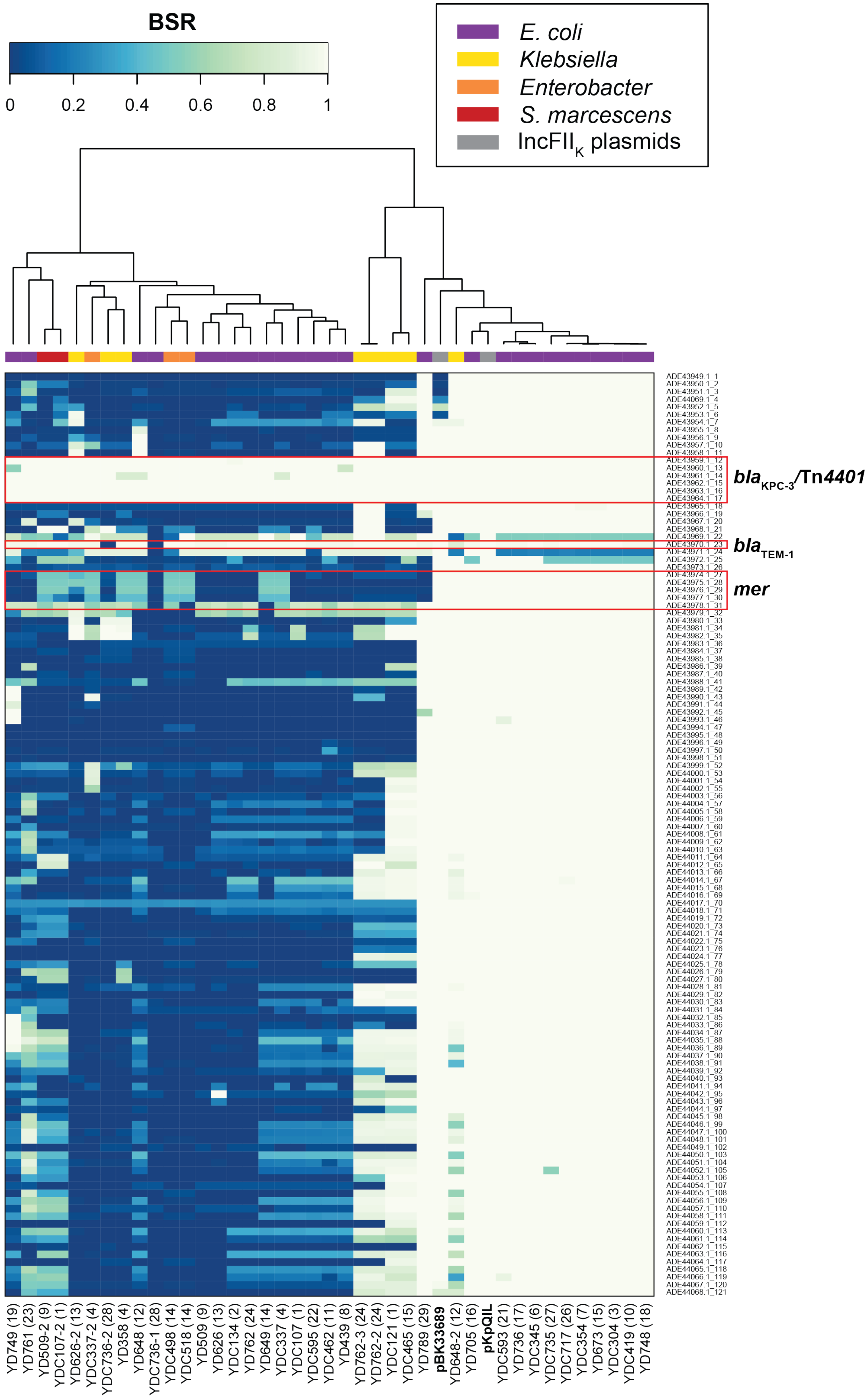
BSR
0
0.2
0.4
0.6
0.8
1
E. coli
Klebsiella
Enterobacter
S. marcescens
IncFII$_K$ plasmids
bla$_{KPC-3}$/Tn4401
bla$_{TEM-1}$
mer
YD749 (19)
YD761 (23)
YD509-2 (9)
YDC107-2 (1)
YD626-2 (13)
YDC337-2 (4)
YDC736-2 (28)
YD358 (4)
YD648 (12)
YDC736-1 (28)
YDC498 (14)
YDC518 (14)
YD509 (9)
YD626 (13)
YDC134 (2)
YD762 (24)
YD649 (14)
YDC337 (4)
YDC107 (1)
YDC595 (22)
YDC462 (11)
YD439 (8)
YD762-3 (24)
YD762-2 (24)
YDC121 (1)
YDC465 (15)
YD789 (29)
pBK33689
YD648-2 (12)
YD705 (16)
pKpQIL
YDC593 (21)
YD736 (17)
YDC345 (6)
YDC735 (27)
YDC717 (26)
YDC354 (7)
YD673 (15)
YDC304 (3)
YDC419 (10)
YD748 (18)

Figure S4. *In silico* detection of the $IncFII_K$ plasmid pKpQIL (GenBank accession no. GU595196.1) in the bla_{KPC}-containing *E. coli* and other bacterial species analyzed in this study. The heat map contains BSR values indicating the presence (very light green) or absence (dark blue) of each plasmid gene in each of the genomes. The heat map was constructed with the heatmap.2 function of gplots using R v.3.4.1. Rows represent each of the protein-coding genes of pYDC107_85, while each column represents a different genome. The species and/or genus of each genome is indicated by a square at the top of the heat map (see inset legend). The previously sequenced $IncFII_K$ plasmids pKpQIL (GenBank accession no. GU595196.1) and pBK33689 (Genbank accession no. KU295133.1) are indicated by a purple label and are in bold. Red boxes indicate the locations of the bla_{KPC-3}/Tn*4401* region, bla_{TEM-1}, and mercury resistance genes (*mer*). The patient number (see Table S1) corresponding to each of the strains is indicated in parentheses next to the strain number.

Table S1. Genomes sequenced in this study

Patient and Strain					Genome Characteristics									Genome Assembly Details			
Patient No.	Species	Strain	Year	Strain Source	*E. coli* Molecular Serotype[a]	ST (CC)[b]	*E. coli* Phylogroup[c]	bla_{KPC} Gene	Tn*4401* isoform	Plasmids	Size of bla_{KPC} contig (bp)	Inc-type of bla_{KPC} contig	Similarity with IncN or IncFII$_K$ plasmids[d]	No. of Contigs	Genome Size (Mb)	GC %	Accession No.
1	*E. coli*	YDC107	2008	(Sidjabat *et al.* 2009)	O102:H6	964	D	bla_{KPC-3}	Tn*4401*b	IncI1(AP005147), IncFIA(AP001918), IncFIB(AP001918), IncFII(AY458016), IncN(AY046276)	70,372	IncN	IncN	7	5.65	50.62	CP025707-CP025713
1	*K. pneumoniae*	YDC121	2008	This study	ND	37	ND	bla_{KPC-3}	Tn*4401*b	Col(MG828), IncFII(pKP91), Col(BS512), IncN(AY046276), IncFIB$_K$	15,781	none	IncN, IncFII$_K$-like	123	5.53	57.20	PGID00000000
1	*S. marcescens*	YDC107-2	2009	This study	ND	ND	ND	bla_{KPC-3}	Tn*4401*b	IncHI2A, IncL/M(pMU407), Col(MG828), IncFII(pCRY), IncN(AY046276), ColRNAI, IncFIB$_K$, Col(BS512)	11,719	none	IncN	187	5.78	58.34	PGIC00000000
2	*E. coli*	YDC134	2008	(Kim *et al.* 2012)	O25:H4	131	B2	bla_{KPC-3}	Tn*4401*b	IncI1, Col(BS512), Col(MG828), IncN(AY046276), IncFII(pRSB107), IncFIA(AP001918), IncFIB(AP001918)	12,168	none	IncN	197	5.34	50.73	PGIB00000000
3	*E. coli*	YDC304	2009	(Kim *et al.* 2012)	O25:H4	131	B2	bla_{KPC-2}	Tn*4401*a	IncFIB(pQil), IncFII$_K$, IncFIA(AP001918), Col(BS512), IncFIB(AP001918), IncI1	62,630	IncFII$_K$	IncFII$_K$	156	5.28	50.8	PGIA00000000
4	*E. coli*	YDC337	2009	(Kim *et al.* 2012)	O147:H7	2521	B1	bla_{KPC-3}	Tn*4401*b	Col(MG828), IncFII(29), IncA/C$_2$, IncN(AY046276), Col(BS512)	6,975	none	IncN	135	5.04	50.81	PGHZ00000000
4	*E. asburiae*	YDC337-2	2009	This study	ND	ND	ND	bla_{KPC-3}	Tn*4401*b	IncA/C$_2$, IncHI1B(CIT), IncFII(pECLA), IncFIB$_K$, Col(BS512), IncN(AY046276), IncHI1A(CIT)	10,113	none	IncN	134	5.21	55.20	PGIQ00000000
4	*K. michiganensis*	YD358	2009	This study	ND	ND	ND	bla_{KPC-3}	Tn*4401*b	Col(MG828), *repA*(pKPC-CAV1321), IncN(AY046276), Col(BS512), IncA/C$_2$	6,975	none	IncN	163	6.66	55.46	PGIP00000000
6	*E. coli*	YDC345	2009	(Kim *et al.* 2012)	O25:H4	131	B2	bla_{KPC-2}	Tn*4401*a	IncFIB(pQil), IncFII$_K$, IncFII(AY458016), Col(BS512)	63,342	IncFII$_K$	IncFII$_K$	140	5.28	50.71	PGHY00000000
7	*E. coli*	YDC354	2009	(Kim *et al.* 2012)	O25:H4	131	B2	bla_{KPC-2}	Tn*4401*a	IncFIB(pQil), IncFII(pRSB107), IncFIA(AP001918), IncX4, Col(BS512), IncFIB(AP001918)	9,907	none	IncFII$_K$	151	5.26	50.77	PGHX00000000
8	*E. coli*	YD439	2009	(Kim *et al.* 2012)	O25:H4	131	B2	bla_{KPC-3}	Tn*4401*b	IncFII(pRSB107), Col(MG828), IncI1(AP005147), IncFIB(AP001918), p0111(AP010962), IncFIA(AP001918), IncN(AY046276), Col(BS512)	11,087	none	IncN	145	5.28	50.72	PGHW00000000
9	*E. coli*	YD509	2010	(Kim *et al.* 2012)	O18ac:H31	372	B2	bla_{KPC-3}	Tn*4401*b	Col(BS512), *repA*(pKPC-2)	10,006	none	none	84	4.81	50.44	PGHV00000000
9	*S. marcescens*	YD509-2	2010	This study	ND	ND	ND	bla_{KPC-3}	Tn*4401*b	IncL/M, IncHI2A, Col(BS512), IncFIB$_K$, IncFII(pCRY)	11,719	none	none	168	5.79	58.17	PGHU00000000
10	*E. coli*	YDC419	2010	(Kim *et al.* 2012)	O25:H4	131	B2	bla_{KPC-2}	Tn*4401*a	ColRNAI, IncFII(pRSB107), Col(BS512), IncI1(AP005147), IncFII$_K$, IncFIB(pQil), IncFIB(AP001918), IncFIA(AP001918)	62,630	IncFII$_K$	IncFII$_K$	148	5.32	50.84	PGHT00000000
11	*E. coli*	YDC462	2011	(Kim *et al.* 2012)	O25:H4	131	B2	bla_{KPC-3}	Tn*4401*b	Col(BS512), IncFIA(AP001918), IncFIB(AP001918), IncFII(AY458016), IncFII(pRSB107)	12,357	none	none	168	5.12	50.75	PGHS00000000
12	*E. coli*	YD648	2011	(Kim *et al.* 2012)	O147:H7	2521	B1	bla_{KPC-3}	Tn*4401*b	IncFII(29), IncFIB(AP001918), IncA/C$_2$, IncN(AY046276), Col156, Col(BS512), IncX1	6,975	none	IncN	185	5.11	50.73	PGHR00000000
12	*K. pneumoniae*	YD648-2	2011	This study	ND	258	ND	bla_{KPC-2}	Tn*4401*a	Col(MG828), IncR, IncFII$_K$, ColRNAI, IncX3, IncFIB(pQil), Col(BS512)	20,615	none	IncFII$_K$	147	5.55	57.20	PGHQ00000000
13	*E. coli*	YD626	2011	(Kim *et al.* 2012)	O1:H6	648	F	bla_{KPC-2}*	Tn*4401*b*	Col(BS512), IncN(AY046276)	20,704	none	IncN	138	5.26	50.52	PGHP00000000
13	*K. variicola*	YD626-2	2011	This study	ND	454	ND	bla_{KPC-2}*	Tn*4401*b*	IncFIA(pBK30683), IncHI1B(JN420336), IncFII$_K$, IncN(AY046276), Col(BS512)	20,494	none	IncN	154	5.83	57.03	PGIO00000000
14	*E. coli*	YD649	2011	(O'Hara *et al.* 2014)	ONT:H49	5743	B1	bla_{KPC-3}	Tn*4401*b	IncFII(29), IncFIB(AP001918), IncA/C$_2$, Col(MG828), IncI2, Col(BS512)	10,006	none	none	91	5.05	50.76	PGHO00000000
14	*E. hormaechei*	YDC498	2011	This study	ND	ND	ND	bla_{KPC-3}	Tn*4401*b	IncA/C$_2$, Col(BS512)	14,153	none	none	81	4.64	55.19	PGIN00000000
14	*E. hormaechei*	YDC518	2011	This study	ND	ND	ND	bla_{KPC-3}	Tn*4401*b	IncA/C$_2$, Col(MG828), Col(BS512)	14,167	none	none	92	4.62	55.21	PGIM00000000
15	*E. coli*	YD673	2011	(O'Hara *et al.* 2014)	O25:H4	131	B2	bla_{KPC-2}	Tn*4401*a	IncFIB(pQil), Col(BS512), IncFIB(AP001918), IncFIA(AP001918), IncFII$_K$, IncFII(pRSB107), IncN(AY046276)	9,907	none	IncN, IncFII$_K$	170	5.32	50.82	PGHN00000000
15	*K. pneumoniae*	YDC465	2011	This study	ND	37	ND	bla_{KPC-3}	Tn*4401*b	Col(BS512), Col(MG828), IncFII(pKP91), IncX3, IncFIB$_K$, ColRNAI, IncN(AY046276)	10,006	none	IncN, IncFII$_K$-like	126	5.57	57.12	PGHM00000000
16	*E. coli*	YD705	2011	(O'Hara *et al.* 2014)	O25:H4	131	B2	bla_{KPC-2}	Tn*4401*a	IncFIB(pQil), IncFII(pRSB107), Col(BS512), Col156, IncFII$_K$, IncFIA(AP001918), IncFIB(AP001918)	62,630	IncFII$_K$	IncFII$_K$	160	5.32	50.82	PGHL00000000
17	*E. coli*	YD736	2012	(O'Hara *et al.* 2014)	O25:H4	131	B2	bla_{KPC-2}	Tn*4401*a	IncI1, IncFIB(pQil), IncFII$_K$, IncFIA(AP001918), Col(BS512)	18,604	none	IncFII$_K$	136	5.2	50.78	PGHK00000000
18	*E. coli*	YD748	2012	(O'Hara *et al.* 2014)	O25:H4	131	B2	bla_{KPC-2}	Tn*4401*a	Col(BS512), IncFII$_K$, IncFIB(pQil), IncFIA(AP001918), IncFII(pRSB107), IncFIB(AP001918)	62,630	IncFII$_K$	IncFII$_K$	147	5.19	50.85	PGHJ00000000
19	*E. coli*	YD749	2012	(O'Hara *et al.* 2014)	O25:H4	131	B2	bla_{KPC-3}	Tn*4401*b	Col(BS512), IncI1, IncFII(pHN7A8), IncN(AY046276), IncFIA(AP001918), ColRNAI, IncFIB(AP001918)	6,466	none	IncN	232	5.36	50.79	PGHI00000000
21	*E. coli*	YDC593	2012	This study	O25:H4	131	B2	bla_{KPC-2}	Tn*4401*a	IncFII$_K$, IncI1, Col(BS512), IncFIA(AP001918), IncFII(pRSB107), IncFIB(pQil), IncFIB(AP001918)	31,972	IncFII$_K$	IncFII$_K$	149	5.27	50.81	PGHH00000000
22	*E. coli*	YDC595	2013	This study	O50/O2:H7	95 (95)	B2	bla_{KPC-2}*	Tn*4401*b*	Col(MG828), IncFII(29), Col156, Col(BS512), ColRNAI, IncFIB(AP001918)	22,053	none	none	104	5.1	50.60	PGHG00000000
23	*E. coli*	YD761	2014	This study	O15:H18	69 (69)	D	bla_{KPC-3}	Tn*4401*b	Col(BS512), IncFII(CP000670)	78,880	IncFII(CP000670)	none	118	5.1	50.64	PGHF00000000
24	*E. coli*	YD762	2014	This study	O16:H5	131	B2	bla_{KPC-3}	Tn*4401*b	IncFIA(AP001918), Col(MG828), IncFII(pRSB107), Col(BS512), Col156, IncN(AY046276), IncFIB(pB171)	11,026	none	IncN	124	4.91	50.77	PGHE00000000
24	*K. pneumoniae*	YD762-2	2015	This study	ND	258	ND	bla_{KPC-2}	Tn*4401*a	IncX3, IncFIB$_K$, IncFII$_K$, Col(BS512), IncN(AY046276), IncR, ColRNAI	64,566	IncFII$_K$	IncFII$_K$-like	132	5.73	56.99	PGHD00000000
24	*K. pneumoniae*	YD762-3	2014	This study	ND	258	ND	bla_{KPC-2}	Tn*4401*a	IncR, ColRNAI, IncX3, IncN(AY046276), IncFIB$_K$, IncFII$_K$, Col(BS512)	64,567	IncFII$_K$	IncFII$_K$-like	149	5.74	56.99	PGHC00000000
26	*E. coli*	YDC717	2015	This study	O25:H4	131	B2	bla_{KPC-2}	Tn*4401*a	IncFII(pRSB107), IncFII$_K$, IncFIA(AP001918), Col(BS512), ColRNAI, IncFIB(AP001918)	62,630	IncFII$_K$	IncFII$_K$	148	5.17	50.82	PGHB00000000
27	*E. coli*	YDC735	2015	This study	O25:H4	131	B2	bla_{KPC-2}	Tn*4401*a	IncFIA(AP001918), IncFII$_K$, IncFIB(pQil), IncFII(pRSB107), Col(BS512), IncFIB(AP001918), Col(MG828)	45,152	IncFII$_K$	IncFII$_K$	136	5.22	50.85	PGHA00000000
28	*E. coli*	YDC736-1	2015	This study	O75:H5	1858	B2	bla_{KPC-2}*	Tn*4401*b*	none	15,997	none	none	62	4.84	50.48	PGGZ00000000
28	*K. michiganensis*	YDC736-2	2015	This study	ND	ND	ND	bla_{KPC-2}*	Tn*4401*b*	IncN(AY046276), ColRNAI, IncFIB$_K$	14,471	none	IncN	94	6.09	55.98	PGIL00000000
29	*E. coli*	YD789	2015	This study	O25:H4	131	B2	bla_{KPC-2}	Tn*4401*a	IncFII$_K$, IncFIA(AP001918), IncFII(AY458016), IncFIB(pQil)	57,798	IncFII$_K$	IncFII$_K$	130	5.14	50.80	PGGY00000000
29	*P. stuartii*	YD789-2	2015	This study	ND	ND	ND	none	none	Col156	none	none	none	56	4.36	41.28	PGGX00000000

[a]Molecular serotypes were predicted for the *E. coli* genomes. ND indicates that a serotype was not applicable for the other bacterial species analyzed.
[b]Multilocus sequence typing (MLST) sequence types (STs) were determined for each *E. coli* genome according to the MLST scheme developed by Wirth *et al.* (2006). The *K. pneumoniae* STs were determined using the Institut Pasteur website (http://bigsdb.pasteur.fr). The clonal complex (CC) is in parentheses.
[c]The phylogroup that each of the *E. coli* genomes occupied in the whole-genome phylogeny. ND indicates a phylogroup determination was not determined because the strain is a species other than *E. coli*.
[d]IncN and IncFIIK indicate that genes with similarity to the IncN plasmid pYDC107_70 (GenBank accession no. CP025710.1) and/or IncFII$_K$ plasmid pKpQIL (GenBank accession no. GU595196.1) were identified in the genomes.

Table S2. Minimum inhibitory concentrations (MICs) of the bacterial strains analyzed in this study

Patient No.	Species[a]	Strain	ST (CC)[c]	bla_{KPC} Gene	Other β-lactamase Genes[d]	Other Resistance Genes[d]	MIC (µg/ml)[a]																				
							AMK	TIM	ATM	TZP	SXT	GEN	TOB	CIP	LVX	DOX	MIN	FEP	CAZ	CTX	TGC	ETP	DOR	IPM	MEM	CST	POL
1	*E. coli*	YDC107	964	bla_{KPC-3}	bla_{OXA-9}, bla_{TEM-1} (2 copies), bla_{CMY-44},	*sul2, aph(6)-Id, dfrA14, aadA, aac(6')-Ib, aph(3'')-Ib, mrx/mphA, aadA5, tet(A), tet(D), aac(3)-IIa, sul1, aph(3'')-Ib, ermB*	≤4	≤16/2	8	≤8/4	4/76	≤1	≤1	1	≤1	8	≤2	≤2	8	4	≤0.25	>4	2	8	8	≤0.25	≤0.25
1	*K. pneumoniae*	YDC121	37	bla_{KPC-3}	bla_{OXA-9}, bla_{TEM-1},bla_{SHV-11}	*aadA, aph(3'')-Ib, aph(6)-Id, sul2, aac(6')-Ib, fosA5*	≤4	>128/2	>16	>64/4	>4/76	>8	>8	>2	>8	4	4	>16	>16	>32	0.5	>4	>2	>8	>8	≤0.25	≤0.25
1	*S. marcescens*	YDC107-2	ND	bla_{KPC-3}	bla_{OXA-9}, bla_{TEM-1}, bla_{SRT-2}, bla_{OXA-2}	*aadA, aph(3'')-Ib, aph(6)-Id, sul2, aac(6')-Ib, sul1, aadA11, ant(2'')-Ia, aac(6')-Ic, dfrB3*	8	>128/2	>16	64/4	>4/76	>8	>8	>2	4	4	4	>16	>16	>32	0.5	>4	>2	>8	>8	>4	>4
2	*E. coli*	YDC134	131	bla_{KPC-3}	bla_{TEM-1}	*aph(3'')-Ib, aph(6)-Id, sul2, dfrA14*	≤4	>128/2	>16	64/4	>4/76	≤1	≤1	>2	>8	8	≤2	4	>16	8	≤0.25	0.5	1	≤1	≤1	≤0.25	≤ 0.25
3	*E. coli*	YDC304	131	bla_{KPC-2}	bla_{TEM-1}, bla_{OXA-9}	*aadA, aadA5, aph(3'')-Ib, aph(6)-Id, mphA/mrx, sul1, sul2, aac(6')-Ib, dfrA17*	≤4	>128/2	>16	64/4	>4/76	≤1	≤1	>2	>8	16	8	≤2	8	4	≤0.25	0.5	0.25	≤1	≤1	≤0.25	≤ 0.25
4	*E. coli*	YDC337	2521	bla_{KPC-3}	bla_{OXA-9}, bla_{TEM-1}, bla_{SHV-7}	*aadA, sul1, qnrA1, aac(6')-Ib, ant(2'')-Ia, dfrA14*	≤4	>128/2	>16	32/4	>4/76	>8	>8	0.5	≤1	≤2	≤2	≤2	16	4	≤0.25	≤0.25	≤ 0.12	≤1	≤1	≤0.25	≤ 0.25
4	*E. asburiae*	YDC337-2	ND	bla_{KPC-3}	bla_{OXA-9}, bla_{SHV-5}, bla_{ACT-3}, $bla_{TEM-206}$	*aadA, sul1, qnrA1, ant(2'')-Ia, aac(6')-Ib3, aac(1), cat, fosA2, dfrA25*	≤4	>128/2	>16	>64/4	>4/76	>8	>8	>2	>8	4	4	>16	>16	>32	0.5	>4	>2	>8	>8	≤0.25	≤0.25
4	*K. michiganensis*	YD358	ND	bla_{KPC-3}	bla_{OXA-9}, bla_{TEM-1}, bla_{SHV-7}, $bla_{OXY-1-2}$	*aadA, aph(3')-Ia, sul1, aac(6')-Ib, ant(2'')-Ia, dfrA14, fosA5*	≤4	>128/2	>16	32/4	>4/76	≤1	>8	2	4	4	4	>16	>16	>32	0.5	>4	>2	>8	8	>4	>4
6	*E. coli*	YDC345	131	bla_{KPC-2}	bla_{TEM-1}, bla_{OXA-1}, bla_{OXA-9}	*aadA5, sul1, catB3, aac(6')-Ib-cr, dfrA14, dfrA17*	8	>128/2	>16	>64/4	>4/76	≤1	>8	>2	>8	≤2	≤2	≤2	2	4	≤0.25	0.5	0.25	≤1	≤1	≤0.25	≤0.25
7	*E. coli*	YDC354	131	bla_{KPC-2}	bla_{TEM-1}, bla_{OXA-9}	*aadA, aadA5, aph(3'')-Ib, aph(6)-Id, mphA/mrx, sul1, sul2, aac(6')-Ib, dfrA17*	≤4	>128/2	>16	>64/4	>4/76	≤1	≤1	>2	>8	8	≤2	8	8	8	≤0.25	1	2	2	4	≤0.25	≤0.25
8	*E. coli*	YD439	131	bla_{KPC-3}	bla_{TEM-1}, bla_{OXA-9}	*aadA, aac(6')-Ib, dfrA14*	≤4	>128/2	>16	>64/4	>4/76	≤1	≤1	>2	>8	≤2	≤2	4	16	8	≤0.25	1	0.5	≤1	≤1	≤0.25	≤0.25
9	*E. coli*	YD509	372	bla_{KPC-3}	bla_{TEM-1}	*aadA, sat-1, dfrA25, cat*	≤4	>128/2	>16	32/4	>4/76	≤1	≤1	≤0.25	≤1	≤2	≤2	8	8	8	≤0.25	0.5	0.25	≤1	≤1	≤0.25	≤0.25
9	*S. marcescens*	YD509-2	ND	bla_{KPC-3}	bla_{OXA-2}, bla_{OXA-9}, bla_{TEM-1}, bla_{SHV-7}, bla_{SRT-2}	*aadA, aadA11, aph(3'')-Ib, aph(6)-Id, sul1, sul2, aac(6')-Ib, ant(2'')-Ia, aac(6')-Ic, dfrB3*	≤4	>128/2	>16	>64/4	>4/76	>8	>8	≤0.25	≤1	4	4	4	>16	16	0.5	1	1	2	≤1	≤0.25	≤0.25
10	*E. coli*	YDC419	131	bla_{KPC-2}	bla_{TEM-1}, bla_{OXA-9}	*aac(3)-Iia, aadA, aadA5, aph(3'')-Ib, aph(6)-Id, mphA/mrx, sul1, sul2, aac(6')-Ib, dfrA17*	≤4	>128/2	>16	64/4	>4/76	>8	8	>2	8	8	≤2	≤2	4	2	≤0.25	0.5	0.25	≤1	≤1	≤0.25	≤0.25
11	*E. coli*	YDC462	131	bla_{KPC-3}	bla_{TEM-1}	*aadA5, aph(3'')-Ib, aph(6)-Id, mphA/mrx, sul1, sul2, dfrA17*	≤4	>128/2	>16	>64/4	>4/76	≤1	≤1	>2	>8	8	≤2	8	>16	16	≤0.25	1	0.5	≤1	≤1	≤0.25	≤0.25
12	*E. coli*	YD648	2521	bla_{KPC-3}	bla_{OXA-9}, bla_{TEM-1}, bla_{SHV-7}	*aadA, sul1, qnrA1, aac(6')-Ib, ant(2'')-Ia, dfrA14*	≤4	>128/2	>16	32/4	>4/76	≤1	2	≤0.25	≤1	≤2	≤2	≤2	≤ 1	2	≤0.25	0.5	0.25	≤1	≤1	≤0.25	≤0.25
12	*K. pneumoniae*	YD648-2	258	bla_{KPC-2}	$bla_{SHV-160}$, bla_{OXA-9}	*aadA2, mphA/mrx, sul1, catI, aac(6')-Ib, dfrA12, fosA5*	>32	>128/2	>16	>64/4	>4/76	4	>8	>2	>8	4	4	>16	>16	>32	0.5	>4	>2	>8	>8	≤0.25	≤0.25
13	*E. coli*	YD626	648	bla_{KPC-2}	bla_{TEM-1}, $bla_{SHV-160}$, bla_{LAP-1}	*qnrS1, dfrA14*	≤4	>128/2	>16	>64/4	4/76	≤1	≤1	>2	>8	≤2	≤2	4	>16	16	≤0.25	2	0.5	2	2	≤0.25	≤0.25
13	*K. variicola*	YD626-2	ND	bla_{KPC-2}	bla_{TEM-1}, $bla_{SHV-160}$, bla_{LAP-1}, bla_{LEN-2}	*qnrS1, fosA5*	≤4	>128/2	>16	>64/4	>4/76	≤1	2	>2	>8	4	≤2	4	>16	16	≤0.25	4	1	≤1	2	≤0.25	≤0.25
14	*E. coli*	YD649	5743	bla_{KPC-3}	bla_{OXA-9}, bla_{TEM-1}, bla_{SHV-7}	*aadA, sul1, qnrA1, aac(6')-Ib, ant(2'')-Ia, dfrA14*	≤4	>128/2	>16	32/4	≤0.5/9.5	>8	>8	1	≤1	4	4	≤2	>16	4	≤0.25	≤ 0.25	≤ 0.12	≤1	≤1	≤0.25	≤0.25
14	*E. hormaechei*	YDC498	ND	bla_{KPC-3}	bla_{OXA-9}, bla_{ACT-37}, bla_{SHV-7}, bla_{TEM-1}	*aadA, sul1, qnrA1, aac(6')-Ib, ant(2'')-Ia, cat*	≤4	>128/2	>16	32/4	2/38	>8	>8	2	2	≤2	≤2	4	>16	16	≤0.25	1	0.5	≤0.5	≤1	≤0.25	≤0.25
14	*E. hormaechei*	YDC518	ND	bla_{KPC-3}	bla_{OXA-9}, bla_{ACT-37}, bla_{SHV-7}, bla_{TEM-1}	*aadA, sul1, qnrA1, aac(6')-Ib, ant(2'')-Ia, cat*	16	>128/2	>16	32/4	2/38	>8	>8	>2	8	4	4	4	>16	32	≤0.25	0.5	1	≤0.5	>8	≤0.25	≤0.25
15	*E. coli*	YD673	131	bla_{KPC-2}	bla_{TEM-1}, bla_{OXA-9}	*aac(3)-IIa, aadA5, aph(3'')-Ib, aph(6)-Id, mphA/mrx, sul1, sul2, aac(6')-Ib, dfrA14, dfrA17*	≤4	>128/2	>16	32/4	>4/76	>8	8	>2	8	8	≤2	≤2	4	2	≤0.25	0.5	0.25	≤1	≤1	≤0.25	≤0.25
15	*K. pneumoniae*	YDC465	ND	bla_{KPC-3}	bla_{OXA-9}, bla_{SHV-11}, bla_{TEM-1}	*aadA, aph(3'')-Ib, aph(6)-Id, sul2, aac(6')-Ib, fosA5*	32	>128/2	>16	32/4	>4/76	≤1	>8	>2	>8	≤2	≤2	>16	>16	>32	≤0.25	>4	>2	8	>8	≤0.25	≤0.25
16	*E. coli*	YD705	131	bla_{KPC-2}	$bla_{TEM-206}$, bla_{OXA-9}	*aac(3)-IIa, aadA5, aph(3'')-Ib, aph(6)-Id, mphA/mrx, sul1, sul2, dfrA17*	≤4	>128/2	>16	32/4	>4/76	>8	>8	>2	>8	16	≤2	≤2	4	4	≤0.25	1	0.25	≤1	≤1	≤0.25	≤0.25
17	*E. coli*	YD736	131	bla_{KPC-2}	bla_{TEM-12}, bla_{OXA-9}	*aadA, aac(6')-Ib, dfrA14*	≤4	>128/2	>16	>64/4	≤0.5/9.5	≤1	≤1	>2	>8	≤2	≤2	8	16	16	≤0.25	2	1	≤1	2	≤0.25	≤0.25
18	*E. coli*	YD748	131	bla_{KPC-2}	bla_{TEM-1}, bla_{OXA-9}	*aac(3)-IIa, aadA5, aph(3'')-Ib, aph(6)-Id, mphA/mrx, sul1, sul2, dfrA17*	≤4	>128/2	8	16/4	>4/76	>8	8	>2	>8	8	≤2	≤2	≤1	≤ 1	≤0.25	≤ 0.25	≤ 0.12	≤1	≤1	≤0.25	≤0.25
19	*E. coli*	YD749	131	bla_{KPC-3}	$bla_{CTX-M-14}$, bla_{OXA-9}, bla_{TEM-1}	*aadA, aadA5, aph(3'')-Ib, aph(6)-Id, mphA/mrx, sul1, sul2, aac(6')-Ib, dfrA14, dfrA17*	≤4	>128/2	>16	>64/4	>4/76	≤1	≤1	>2	8	16	≤2	>16	>16	>32	≤0.25	4	2	2	2	≤0.25	≤0.25
21	*E. coli*	YDC593	131	bla_{KPC-2}	bla_{TEM-1}, bla_{OXA-9}	*aac(3)-Iia, aadA5, sul1, aadA24, dfrA14, dfrA17, aac(6')-Iid*	≤4	>128/2	16	32/4	>4/76	>8	8	>2	>8	≤2	≤2	≤2	8	2	≤0.25	0.5	0.25	≤0.5	≤1	≤0.25	≤0.25
22	*E. coli*	YDC595	95 (95)	bla_{KPC-2}	bla_{TEM-1}	none	≤4	>128/2	>16	>64/4	≤0.5/9.5	≤1	≤1	≤0.25	≤1	≤2	≤2	8	8	16	≤0.25	4	1	2	2	0.5	≤0.25
23	*E. coli*	YD761	69 (69)	bla_{KPC-3}	bla_{TEM-1}, $bla_{SHV-160}$	*aac(6')-IId*	≤4	128/2	>16	≤8/4	≤0.5/9.5	≤1	≤1	≤0.25	≤1	≤2	≤2	≤2	8	4	≤0.25	≤0.25	≤0.12	≤1	≤1	≤0.25	≤0.25
24	*E. coli*	YD762	131	bla_{KPC-3}	bla_{TEM-1}, bla_{OXA-9}	*aadA, aph(3'')-Ib, aph(6)-Id, sul1, sul2, aac(6')-Ib, dfrA14*	16	>128/2	>16	>64/4	>4/76	>8	>8	≤0.25	≤1	≤2	≤2	4	>16	8	≤0.25	0.5	0.5	2	≤1	≤0.25	≤0.25
24	*K. pneumoniae*	YD762-2	258	bla_{KPC-2}	bla_{TEM-1}, $bla_{SHV-160}$, bla_{OXA-9}	*aac(3)-IV, aadA, aadA2, aph(4)-Ia, sul3, aac(6')-Ib, fosA5*	16	>128/2	>16	>64/4	≤0.5/9.5	>8	>8	>2	>8	≤2	≤2	4	>16	16	≤0.25	2	1	≤1	≤1	≤0.25	≤0.25
24	*K. pneumoniae*	YD762-3	258	bla_{KPC-2}	bla_{TEM-1}, $bla_{SHV-160}$, bla_{OXA-9}	*aadA, aadA2, aph(4)-Ia, sul3, aac(6')-Ib, fosA5*	32	>128/2	>16	>64/4	0.5/9.5	≤1	>8	>2	>8	≤2	≤2	8	>16	32	≤0.25	>4	1	2	4	≤0.25	≤0.25
26	*E. coli*	YDC717	131	bla_{KPC-2}	bla_{TEM-1}, $bla_{CTX-M-27}$, bla_{OXA-9}	*aadA5, aph(3'')-Ib, aph(6)-Id, mphA/mrx, sul1, sul2, aac(6')-Ib, dfrA17*	≤4	>128/2	>16	32/4	≤0.5/9.5	≤1	≤1	>2	>8	≤2	≤2	8	8	8	≤0.25	4	1	2	2	0.5	0.5
27	*E. coli*	YDC735	131	bla_{KPC-2}	bla_{TEM-1}, bla_{OXA-9}	*aac(3)-IIa, aadA5, aph(3'')-Ib, aph(6)-Id, mphA/mrx, sul1, sul2, aadA24, dfrA17*	≤4	>128/2	>16	>64/4	>4/76	>8	8	>2	>8	>8	8	≤2	4	4	≤0.25	1	0.25	2	≤1	≤0.25	1
28	*E. coli*	YDC736-1	1858	bla_{KPC-2}	none	none	≤4	>128/2	>16	>64/4	≤0.5/9.5	≤1	≤1	0.5	≤1	≤2	≤2	4	4	8	≤0.25	1	0.5	≤1	≤1	≤0.25	≤0.25
28	*K. michiganensis*	YDC736-2	ND	bla_{KPC-2}	$bla_{OXY-1-4}$	*qnrB19, dfrA14, fosA5*	≤4	128/2	16	64/4	4/76	≤1	≤1	0.5	≤1	≤2	≤2	≤2	4	2	≤0.25	1	0.5	2	≤1	≤0.25	≤0.25
29	*E. coli*	YD789	131	bla_{KPC-2}	bla_{TEM-1}	none	≤4	>128/2	>16	32/4	≤0.5/9.5	≤1	≤1	0.5	≤1	≤2	≤2	≤2	4	2	≤0.25	1	0.5	2	≤1	≤0.25	≤0.25
29	*P. stuartii*	YD789-2	ND	none	bla_{SRT-2}	*catIII, aac(2')-Ia*	Not tested																				

[a]The antibiotics are abbreviated as follows: amikacin, AMK; ticarcillin/clavulanic acid, TIM; aztreonam, ATM; piperacillin/tazobactam, TZP; trimethoprim-sulfamethoxazole, SXT; gentamicin, GEN; cefepime, FEP; tobramycin, TOB; levofloxacin, LVX; doxycycline, DOX; ciprofloxacin, CIP; minocycline, MIN; meropenem, MEM; cefotaxime, CTX; tigecycline, TGC; ertapenem, ETP; imipenem, IPM; doripenem, DOR; colistin, CST; polymyxin B, POL; and ceftazidime, CAZ. The susceptibility levels are color-coded as resistant (red), intermediate (yellow), or sensitive (green). For cefepime, yellow indicates dose-dependent susceptibility.

[b]Molecular serotypes were predicted for each of the *E. coli* genome assemblies.

[c]Multilocus sequence typing (MLST) sequence types (STs) of *E. coli* and *K. pneumoniae* were determined by analysis of the genome assemblies. The clonal complex (CC) is in parentheses.

[d]β-lactamase genes and other antibiotic resistance genes were identified in each of the genome assemblies. The bla_{CMY} gene identified on the plasmid of YDC107 is listed; however, other bla_{CMY} genes identified in the *E. coli* genomes are not indicated since they exhibited similarity with the *E. coli* chromosomal AmpC β-lactamase gen

Table S3. *bla*$_{KPC}$-containing *E. coli* genomes analyzed in this study

Strain ID	Molecular Serotype[a]	ST (CC)[b]	*bla*$_{KPC}$ Gene	GenBank Accession No.
YDC107	O102:H6	964	*bla*$_{KPC-3}$	CP025707-CP025713
YDC134	O25:H4	131	*bla*$_{KPC-3}$	PGIB00000000
YDC304	O25:H4	131	*bla*$_{KPC-2}$	PGIA00000000
YDC337	O147:H7	2521	*bla*$_{KPC-3}$	PGHZ00000000
YDC345	O25:H4	131	*bla*$_{KPC-2}$	PGHY00000000
YDC354	O25:H4	131	*bla*$_{KPC-2}$	PGHX00000000
YD439	O25:H4	131	*bla*$_{KPC-3}$	PGHW00000000
YD509	O18ac:H31	372	*bla*$_{KPC-3}$	PGHV00000000
YDC419	O25:H4	131	*bla*$_{KPC-2}$	PGHT00000000
YDC462	O25:H4	131	*bla*$_{KPC-3}$	PGHS00000000
YD648	O147:H7	2521	*bla*$_{KPC-3}$	PGHR00000000
YD626	O1:H6	648	*bla*$_{KPC-2}$	PGHP00000000
YD649	ONT:H49	5743	*bla*$_{KPC-3}$	PGHO00000000
YD673	O25:H4	131	*bla*$_{KPC-2}$	PGHN00000000
YD705	O25:H4	131	*bla*$_{KPC-2}$	PGHL00000000
YD736	O25:H4	131	*bla*$_{KPC-2}$	PGHK00000000
YD748	O25:H4	131	*bla*$_{KPC-2}$	PGHJ00000000
YD749	O25:H4	131	*bla*$_{KPC-3}$	PGHI00000000
YDC593	O25:H4	131	*bla*$_{KPC-2}$	PGHH00000000
YDC595	O50/O2:H7	95 (95)	*bla*$_{KPC-2}$	PGHG00000000
YD761	O15:H18	69 (69)	*bla*$_{KPC-3}$	PGHF00000000
YD762	O16:H5	131	*bla*$_{KPC-3}$	PGHE00000000
YDC717	O25:H4	131	*bla*$_{KPC-2}$	PGHB00000000
YDC735	O25:H4	131	*bla*$_{KPC-2}$	PGHA00000000
YDC736-1	O75:H5	1858	*bla*$_{KPC-2}$	PGGZ00000000
YD789	O25:H4	131	*bla*$_{KPC-2}$	PGGY00000000
1326F	O1:H6	648	Contains deletions	GCA_001611725.1
BIDMC102	O102:H6	405 (405)	*bla*$_{KPC-2}$	GCA_001030515.1
BIDMC104	ONT:H8	448 (448)	*bla*$_{KPC-2}$	GCA_001030555.1
BIDMC15	O1:H6	648	*bla*$_{KPC-2}$	GCA_000522245.1
BIDMC17A	O1:H6	648	*bla*$_{KPC-2}$	GCA_000522225.1
BIDMC17B	O1:H6	648	*bla*$_{KPC-2}$	GCA_000522205.1
BIDMC19A	O1:H6	648	*bla*$_{KPC-2}$	GCA_000522185.1
BIDMC19B	O1:H6	648	*bla*$_{KPC-2}$	GCA_000522165.1
BIDMC19C	O1:H6	648	*bla*$_{KPC-2}$	GCA_000474825.1
BIDMC20A	O25:H4	131	*bla*$_{KPC-3}$	GCA_000522145.1
BIDMC20B	O25:H4	131	*bla*$_{KPC-3}$	GCA_000522125.1
BIDMC2B	O1:H6	648	*bla*$_{KPC-2}$	GCA_000522305.1
BIDMC3	O1:H6	648	*bla*$_{KPC-2}$	GCA_000522285.1
BIDMC43a	O1:H6	648	*bla*$_{KPC-2}$	GCA_000522105.1
BIDMC43b	O1:H6	648	*bla*$_{KPC-2}$	GCA_000522085.1
BIDMC58	O25:H4	131	*bla*$_{KPC-3}$	GCA_000692375.1
BIDMC6	O1:H6	648	*bla*$_{KPC-2}$	GCA_000522405.1
BIDMC62	O25:H4	131	*bla*$_{KPC-3}$	GCA_000692415.1
BIDMC64	O25:H4	131	*bla*$_{KPC-3}$	GCA_000692455.1
BIDMC82	O1:H6	648	*bla*$_{KPC-2}$	GCA_000633675.1
BIDMC9	O1:H6	648	*bla*$_{KPC-2}$	GCA_000522265.1
BWH24	O25:H4	131	*bla*$_{KPC-3}$	GCA_000474845.1
BWH34	O25:H4	131	*bla*$_{KPC-3}$	GCA_000522345.1
ECONIH1	O1:H6	648	*bla*$_{KPC-2}$	GCA_000784925.1
ECONIH2	O6:H31	127	*bla*$_{KPC-3}$	GCA_001675145.1
MGH121	O89:H9	167 (10)	*bla*$_{KPC-3}$	GCA_001030425.1
MNCRE44	O25:H4	131	*bla*$_{KPC-3}$	GCA_000931565.1
MNCRE45	O25:H4	131	*bla*$_{KPC-3}$	GCA_000952385.1
MNCRE46	O25:H4	131	*bla*$_{KPC-3}$	GCA_000952305.1
MRSN10204	O83:H1	2554	*bla*$_{KPC-3}$	GCA_000982035.1

[a]Molecular serotypes were predicted for each of the *E. coli* genome assemblies.
[b]Multilocus sequence typing (MLST) sequence types (STs) of *E. coli* and *K. pneumoniae* were determined by analysis of the genome assemblies. The clonal complex (CC) is in parentheses.

Table S4. Annotation of plasmid pYDC107_184

CDS	Locus	Gene	Predicted Protein Function	Start	Stop
1	YDC107_4930	*repA*	RepFIB replication protein A	1	978
2	YDC107_4931		phage integrase family protein	2,003	1,263
3	YDC107_4932		ribbon-helix-helix, CopG family transcriptional regulator	2,917	2,735
4	YDC107_4933		conserved hypothetical protein	2,522	2,779
5	YDC107_4934		putative membrane protein	3,449	4,618
6	YDC107_4935		hypothetical protein	5,107	4,814
7	YDC107_4936		plasmid stabilisation system family protein	5,488	5,213
8	YDC107_4937		ribbon-helix-helix, CopG family transcriptional regulator	5,766	5,488
9	YDC107_4938		putative transcriptional regulator	6,377	7,129
10	YDC107_4939	*kdgT*	2-keto-3-deoxygluconate transporter	8,140	7,175
11	YDC107_4940		L-PSP putative endoribonuclease	8,553	8,173
12	YDC107_4941		dihydrodipicolinate synthetase family protein	9,468	8,578
13	YDC107_4942		putative oxidoreductase	10,082	10,309
14	YDC107_4943		putative sugar transport protein	10,452	11,318
15	YDC107_4944		putative sugar transport protein	11,308	12,195
16	YDC107_4945	*cpdA*	3',5'-cyclic adenosine monophosphate phosphodiesterase CpdA	12,206	13,030
17	YDC107_4946	*ugpC*	sn-glycerol-3-phosphate import ATP-binding protein UgpC	13,036	14,109
18	YDC107_4947		bacterial extracellular solute-binding family protein	14,102	15,412
19	YDC107_4948		transposase	15,836	15,471
20	YDC107_4949		transposase	16,029	15,856
21	YDC107_4950		integrase core domain protein	16,497	16,111
22	YDC107_4951		HTH-like domain protein	16,999	16,541
23	YDC107_4952		transposase IS66 family protein	17,332	18,036
24	YDC107_4953		histidine phosphatase protein	18,624	18,070
25	YDC107_4954		hypothetical protein	19,231	18,746
26	YDC107_4955		thioredoxin-like family protein	19,741	19,256
27	YDC107_4956		ABC transporter family protein	20,423	19,728
28	YDC107_4957		ABC transporter permease	21,540	20,428
29	YDC107_4958		ABC transporter permease	22,831	21,548
30	YDC107_4959		conserved hypothetical protein	24,213	22,834
31	YDC107_4960		periplasmic protein-probably involved in high-affinity Fe2+ transport	24,838	24,317
32	YDC107_4961		iron permease FTR1 family protein	26,801	24,885
33	YDC107_4962	*nqrC*	ubiquinone oxidoreductase, Na(+)-translocating, C subunit	27,879	27,118
34	YDC107_4963		thioredoxin-like family protein	28,622	28,116
35	YDC107_4964		DSBA-like thioredoxin domain protein	28,770	28,612
36	YDC107_4965		hypothetical protein	29,481	29,266
37	YDC107_4966		putative transposase	29,553	29,930
38	YDC107_4967		transposase DDE domain protein	30,395	31,108
39	YDC107_4968	*tetD*	transposon Tn10 TetD protein	31,534	31,118
40	YDC107_4969	*tetC*	transposon Tn10 TetC protein	31,622	32,215
41	YDC107_4970	*tetA*	tetracycline resistance protein, class B	33,533	32,328
42	YDC107_4971		transcriptional regulator	34,902	34,216
43	YDC107_4972	*tetR*	tetracycline repressor protein TetR	33,615	34,238
44	YDC107_4973		amino acid binding protein	35,296	34,910
45	YDC107_4974		putative antibiotic biosynthesis monooxygenase	35,462	35,289
46	YDC107_4975		putative transposase	35,926	35,549
47	YDC107_4976		hypothetical protein	35,998	36,213
48	YDC107_4977	*aadA5*	nucleotidyltransferase domain protein	36,765	37,553
49	YDC107_4978	*qacEΔ1*	small multidrug resistance family protein	37,759	38,106
50	YDC107_4979	*sul1*	dihydropteroate synthase	38,100	38,939
51	YDC107_4980		hypothetical protein	39,067	39,270
52	YDC107_4981	*chrA*	chromate transporter, chromate ion transporter family protein	39,426	40,631
53	YDC107_4982		transcriptional regulator	40,642	40,947
54	YDC107_4983	*tnpA*	transposase IS66 family protein	41,174	41,938
55	YDC107_4984	*mphR(A)*	erythromycin resistance repressor protein MphR(A)	43,015	42,431
56	YDC107_4985	*mrx*	major facilitator superfamily protein Mrx	44,253	43,015
57	YDC107_4986	*mph(A)*	phosphotransferase enzyme family protein Mph(A)	45,155	44,250
58	YDC107_4987		transposase IS66 family protein	45,981	45,277
59	YDC107_4988	*ermL*	23S rRNA methylase leader peptide family protein	46,116	46,211
60	YDC107_4989	*ermB*	rRNA adenine N-6-methyltransferase	46,337	47,074
61	YDC107_4990		putative ermAM-like protein	47,079	47,189
62	YDC107_4991		conserved hypothetical protein	47,704	48,153
63	YDC107_4992	*groEL*	GroEL domain protein	48,114	48,455
64	YDC107_4993		transposase family protein	48,682	50,214
65	YDC107_4994		transposase IS66 family protein	51,241	50,537
66	YDC107_4995		conserved hypothetical protein	52,405	51,278
67	YDC107_4996		putative membrane protein	52,683	52,456
68	YDC107_4997		putative ATPase protein	53,922	53,380
69	YDC107_4998	*aac(3)-IIa*	aminoglycoside 3-N-acetyltransferase family protein AAC(3)-IIa	54,795	53,935
70	YDC107_4999		transposase IS66 family protein	55,208	55,912
71	YDC107_5000		resolvase, N terminal domain protein	56,019	56,351
72	YDC107_5001	*bla*$_{TEM-1}$	beta-lactamase TEM	56,534	57,394
73	YDC107_5002	*aph(6)-Id*	aminoglycoside/hydroxyurea antibiotic resistance kinase family protein APH(6)-Id	58,461	57,625
74	YDC107_5003	*aph(3")-Ib*	phosphotransferase enzyme family protein APH(3")-Ib	59,264	58,461
75	YDC107_5004	*sul2*	dihydropteroate synthase	60,140	59,325
76	YDC107_5005		integrase	60,470	60,646
77	YDC107_5006		transposase family protein	61,832	60,828
78	YDC107_5007		transposase	64,877	61,911
79	YDC107_5008		resolvase, N terminal domain protein	65,134	64,880
80	YDC107_5009		transposase IS66 family protein	65,952	65,248
81	YDC107_5010	*dfrA14*	dihydrofolate reductase type 1	66,495	66,022
82	YDC107_5011		integrase	66,651	67,664
83	YDC107_5012		transposase IS66 family protein	68,816	68,112
84	YDC107_5013		transposase	68,867	70,396
85	YDC107_5014		divergent AAA domain protein	72,383	70,611
86	YDC107_5015	*pemK*	mRNA interferase PemK	73,000	72,668
87	YDC107_5016	*pemI*	antitoxin PemI	73,259	73,002
88	YDC107_5017		CAAX protease self-immunity family protein	74,005	73,352
89	YDC107_5018	*repA*	IncFII family plasmid replication initiator RepA	75,801	74,944
90	YDC107_5019	*repB*	replication regulatory RepB family protein	76,361	76,104
91	YDC107_5020		transposase family protein	76,766	77,788
92	YDC107_5021		putative membrane protein	78,260	78,613
93	YDC107_5022		conserved hypothetical protein	79,520	79,026
94	YDC107_5023		putative transposase	79,953	80,330
95	YDC107_5024		conserved hypothetical protein	81,337	80,813
96	YDC107_5025	*argR*	arginine repressor ArgR	82,233	81,757
97	YDC107_5026		C4-dicarboxylate anaerobic carrier family protein	83,720	82,317
98	YDC107_5027	*argF*	ornithine carbamoyltransferase	84,772	83,768
99	YDC107_5028	*arcC*	carbamate kinase	85,768	84,857
100	YDC107_5029	*arcA*	arginine deiminase	86,999	85,779
101	YDC107_5030		pre-toxin domain with VENN motif family protein	87,841	88,086
102	YDC107_5031	*repA*	IncFII RepA family protein	88,766	88,314
103	YDC107_5032	*repB*	replication regulatory RepB family protein	89,326	89,069
104	YDC107_5033		hok/gef family protein	89,759	89,610
105	YDC107_5034		conserved hypothetical protein	90,185	90,003

106	YDC107_5035		conserved hypothetical protein	89,815	90,057
107	YDC107_5036		putative transposase	90,602	90,988
108	YDC107_5037		conserved hypothetical protein	91,501	91,289
109	YDC107_5038	*finO*	fertility inhibition protein	92,194	91,634
110	YDC107_5039	*traX*	type-F conjugative transfer system pilin acetylase TraX	92,995	92,249
111	YDC107_5040	*traI*	conjugative transfer relaxase protein TraI	98,285	93,015
112	YDC107_5041	*traD*	type IV conjugative transfer system coupling protein TraD	100,465	98,285
113	YDC107_5042	*traT*	enterobacterial TraT complement resistance family protein	101,448	100,717
114	YDC107_5043	*traS*	putative TraS	101,972	101,517
115	YDC107_5044	*traG*	TraG-like, N-terminal region family protein	104,794	101,969
116	YDC107_5045	*traH*	conjugal transfer protein TraH	106,164	104,791
117	YDC107_5046	*trbF*	conjugal transfer protein TrbF	106,543	106,151
118	YDC107_5047	*trbJ*	conjugal transfer protein TrbJ	106,810	106,586
119	YDC107_5048	*trbB*	type-F conjugative transfer system pilin assembly thiol-disulfide isomerase TrbB	107,354	106,800
120	YDC107_5049	*traQ*	type-F conjugative transfer system pilin chaperone TraQ	107,625	107,341
121	YDC107_5050	*trbA*	conjugal transfer protein TrbA	108,363	108,022
122	YDC107_5051		putative membrane protein	107,706	108,041
123	YDC107_5052	*traF*	type-F conjugative transfer system pilin assembly protein TraF	109,120	108,377
124	YDC107_5053	*trbE*	conjugal transfer TrbE family protein	109,370	109,113
125	YDC107_5054	*traN*	type-F conjugative transfer system mating-pair stabilization protein TraN	111,205	109,397
126	YDC107_5055	*trbC*	type-F conjugative transfer system pilin assembly protein TrbC	111,840	111,202
127	YDC107_5056	*traU*	TraU family protein	112,841	111,849
128	YDC107_5057	*traW*	type-F conjugative transfer system protein TraW	113,470	112,838
129	YDC107_5058	*trbI*	type-F conjugative transfer system protein TrbI	113,853	113,467
130	YDC107_5059	*traC*	type-IV secretion system protein TraC	116,477	113,850
131	YDC107_5060	*traV*	type IV conjugative transfer system protein TraV	117,508	116,993
132	YDC107_5061	*trbG*	putative TrbG	117,756	117,505
133	YDC107_5062	*trbD*	conjugal transfer protein TrbD	117,910	117,749
134	YDC107_5063	*trbD*	conjugal transfer protein TrbD	118,069	117,932
135	YDC107_5064	*traP*	TraP family protein	118,646	118,056
136	YDC107_5065	*traB*	conjugal transfer protein TraB	120,063	118,636
137	YDC107_5066	*traK*	type-F conjugative transfer system secretin TraK	120,791	120,063
138	YDC107_5067	*traE*	type IV conjugative transfer system protein TraE	121,344	120,778
139	YDC107_5068	*traL*	type IV conjugative transfer system protein TraL	121,677	121,366
140	YDC107_5069	*traA*	type IV conjugative transfer system pilin TraA	122,057	121,692
141	YDC107_5070	*traY*	TraY domain protein	122,315	122,100
142	YDC107_5071	*traJ*	putative protein TraJ	123,098	122,451
143	YDC107_5072	*traM*	relaxosome protein TraM	123,672	123,289
144	YDC107_5073		lytic transglycosylase	124,105	124,614
145	YDC107_5074		conserved hypothetical protein	124,695	124,862
146	YDC107_5075		conserved hypothetical protein	125,731	124,910
147	YDC107_5076	*yubO*	putative YubO protein	126,137	125,850
148	YDC107_5077		conserved hypothetical protein	126,368	126,162
149	YDC107_5078		putative plasmid maintenance protein	127,252	127,040
150	YDC107_5079		ion channel family protein	129,251	128,628
151	YDC107_5080		putative transposase	129,644	129,267
152	YDC107_5081		hypothetical protein	130,367	130,188
153	YDC107_5082		putative glycoporin	131,761	130,367
154	YDC107_5083		sucrose-6-phosphate hydrolase family protein	133,386	131,956
155	YDC107_5084		H+ symporter family protein	134,663	133,386
156	YDC107_5085	*rafA*	alpha-galactosidase	136,852	134,726
157	YDC107_5086	*rafR*	HTH-type transcriptional regulator RafR	137,958	136,948
158	YDC107_5087		phage integrase family protein	138,859	138,116
159	YDC107_5088		transposase family protein	138,965	139,330
160	YDC107_5089		transposase	139,354	140,193
161	YDC107_5090		caspase domain protein	140,178	140,747
162	YDC107_5091		conserved hypothetical protein	141,272	141,841
163	YDC107_5092		putative membrane protein	142,102	141,848
164	YDC107_5093		methyltransferase domain protein	142,691	142,128
165	YDC107_5094		conserved hypothetical protein	144,100	142,739
166	YDC107_5095		putative conserved predicted protein	144,382	144,152
167	YDC107_5096		conserved hypothetical protein	145,608	145,417
168	YDC107_5097		conserved hypothetical protein	146,027	145,605
169	YDC107_5098		antirestriction family protein	146,499	146,074
170	YDC107_5099		conserved hypothetical protein	147,692	146,916
171	YDC107_5100		conserved hypothetical protein	148,177	147,743
172	YDC107_5101		conserved hypothetical protein	148,412	148,191
173	YDC107_5102		DNA methylase family protein	149,096	148,413
174	YDC107_5103		conserved hypothetical protein	150,407	149,481
175	YDC107_5104	*sopB*	plasmid partitioning protein SopB	152,221	151,250
176	YDC107_5105	*sopA*	plasmid partitioning protein SopA	153,387	152,221
177	YDC107_5106	*repE*	replication initiation protein RepE	154,730	153,975
178	YDC107_5107		resolvase	156,310	155,504
179	YDC107_5108	*ccdB*	toxin CcdB	156,616	156,311
180	YDC107_5109	*ccdA*	antitoxin CcdA	156,836	156,618
181	YDC107_5110		conserved hypothetical protein	157,470	157,667
182	YDC107_5111		putative korC protein	157,948	157,664
183	YDC107_5112		conserved hypothetical protein	159,101	157,968
184	YDC107_5113		putative membrane protein	162,483	159,364
185	YDC107_5114		antidote-toxin recognition MazE family protein	162,790	163,020
186	YDC107_5115		PIN domain protein	163,032	163,433
187	YDC107_5116		AAA domain protein	165,733	163,595
188	YDC107_5117		conserved hypothetical protein	166,198	167,193
189	YDC107_5118		conserved hypothetical protein	167,236	168,129
190	YDC107_5119		putative transposase	168,769	168,266
191	YDC107_5120	*hsdR*	type I restriction enzyme EcoR124II R protein	172,293	169,177
192	YDC107_5121		type I restriction modification DNA specificity domain protein	173,659	172,415
193	YDC107_5122	*hsdM*	type I restriction-modification system EcoR124II M subunit	175,212	173,656
194	YDC107_5123	*phd*	antitoxin Phd	175,395	175,616
195	YDC107_5124	*doc*	toxin Doc	175,616	175,996
196	YDC107_5125	*pdcA*	putative PdcA	176,001	176,180
197	YDC107_5126	*pdcB*	putative PdcB	176,208	176,567
198	YDC107_5127		integrase core domain protein	176,530	176,904
199	YDC107_5128	*hsdR*	type I site-specific deoxyribonuclease, HsdR family domain protein	177,171	176,854
200	YDC107_5129		conserved hypothetical protein	178,379	177,399
201	YDC107_5130		hypothetical protein	178,457	178,546
202	YDC107_5131		amino acid permease family protein	178,623	180,026
203	YDC107_5132	*mmuM*	homocysteine S-methyltransferase	180,013	180,945
204	YDC107_5133		ferric transporter	181,287	181,054
205	YDC107_5134		putative transposase	181,776	181,399
206	YDC107_5135		hypothetical protein	181,848	182,063
207	YDC107_5136		conserved hypothetical protein	182,951	182,583
208	YDC107_5137		conserved hypothetical protein	183,313	183,131

Table S5. Annotation of plasmid pYDC107_85

CDS	Locus	Gene	Predicted Protein Function	Start	Stop
1	YDC107_5138		hypothetical protein	68	244
2	YDC107_5139	*pilU*	type IV leader peptidase family protein	1,744	1,088
3	YDC107_5140	*pilT*	transglycosylase SLT domain protein, PilT	2,289	1,729
4	YDC107_5141	*pilS*	PilS N terminal family protein	2,913	2,299
5	YDC107_5142	*pilR*	type II secretion system (T2SS), F family protein	4,015	2,930
6	YDC107_5143	*pilQ*	type II/IV secretion system family protein	5,581	4,028
7	YDC107_5144	*pilP*	type IV pilus biogenesis family protein	6,044	5,592
8	YDC107_5145	*pilO*	pilin accessory family protein	7,326	6,031
9	YDC107_5146	*pilN*	type IVB pilus formation outer membrane protein, R64 PilN family	9,001	7,319
10	YDC107_5147	*pilM*	PilM family Protein	9,452	9,015
11	YDC107_5148	*pilL*	toxin co-regulated pilus biosynthesis Q family protein	10,519	9,452
12	YDC107_5149	*pilK*	putative IncI1 conjugal transfer protein PilK	11,441	10,848
13	YDC107_5150	*traC*	conserved hypothetical protein	12,675	12,271
14	YDC107_5151	*nusG*	transcription termination factor NusG family protein	13,462	12,929
15	YDC107_5152	*traA*	conjugal transfer protein TraA	14,191	13,904
16	YDC107_5153	*repA*	replication initiation protein	15,344	16,375
17	YDC107_5154	*yafA*	putative YafA protein	17,953	17,444
18	YDC107_5155	*yafB*	ProQ/FinO/YafB family protein	18,611	18,009
19	YDC107_5156	*yagA*	putative YagA	18,965	20,311
20	YDC107_5157		hypothetical protein	21,112	20,810
21	YDC107_5158	*parA*	CobQ/CobB/MinD/ParA nucleotide binding domain protein	21,735	21,109
22	YDC107_5159	*impB*	putative ImpB/UmuC DNA polymerase	23,194	21,923
23	YDC107_5160	*impA*	ImpA/UmuD peptidase S24-like family protein	23,631	23,194
24	YDC107_5161	*impC*	ImpC/DinI-like family protein	23,876	23,628
25	YDC107_5162		conserved hypothetical protein	24,270	25,196
26	YDC107_5163		conserved hypothetical protein	25,626	25,291
27	YDC107_5164	*parM*	plasmid segregation protein ParM	25,964	26,944
28	YDC107_5165	*stbB*	plasmid stability family protein, StbB	26,947	27,369
29	YDC107_5166		DNA methylase family protein	27,760	28,443
30	YDC107_5167		conserved hypothetical protein	28,444	28,665
31	YDC107_5168		conserved hypothetical protein	28,679	29,113
32	YDC107_5169	*ycfA*	putative YcfA	29,158	29,928
33	YDC107_5170		conserved hypothetical protein	29,903	30,373
34	YDC107_5171		antirestriction family protein	30,346	30,771
35	YDC107_5172		conserved hypothetical protein	30,818	31,240
36	YDC107_5173	*ychA*	putative YchA	31,237	31,428
37	YDC107_5174	*ssb*	single-stranded DNA-binding family protein	32,197	32,724
38	YDC107_5175		conserved hypothetical protein	32,782	33,015
39	YDC107_5176		ParB/RepB/Spo0J family partition domain protein	33,074	35,032
40	YDC107_5177	*psiB*	protein PsiB	35,085	35,519
41	YDC107_5178	*psiA*	PsiA family protein	35,516	36,235
42	YDC107_5179	*ygaA*	putative YgaA protein	36,232	36,828
43	YDC107_5180	*ardA*	antirestriction family protein ArdA	37,290	37,790
44	YDC107_5181	*ydfA*	putative YdfA	38,519	38,953
45	YDC107_5182	*ydfB*	putative YdfB	39,047	39,313
46	YDC107_5183	*ccgAII*	putative CcgAII	39,490	39,879
47	YDC107_5184		transposase	39,876	40,778
48	YDC107_5185		conserved hypothetical protein	40,840	41,034
49	YDC107_5186	*ygeA*	putative YgeA protein	41,316	41,065
50	YDC107_5187	*ydiA*	methyltransferase domain protein YdiA	41,896	42,744
51	YDC107_5188		conserved hypothetical protein	43,166	42,831
52	YDC107_5189	*nikA*	NikA protein	43,400	43,732

53	YDC107_5190	*nikB*	relaxase/Mobilisation nuclease domain protein NikB	43,743	46,442
54	YDC107_5191	*trbC*	conjugal transfer protein TrbC	48,771	46,480
55	YDC107_5192	*trbB*	conjugal transfer protein TrbB	49,834	48,764
56	YDC107_5193	*trbA*	putative TrbA	51,061	49,853
57	YDC107_5194	*finQ*	putative FinQ	52,150	51,368
58	YDC107_5195	*blc*	outer membrane lipoprotein Blc	53,193	52,660
59	YDC107_5196	*sugE*	quaternary ammonium compound-resistance protein SugE, partial	52,346	52,663
60	YDC107_5197	bla_{CMY-44}	beta-lactamase CMY-44	54,420	53,287
61	YDC107_5198		transposase DDE domain group 1 family protein	56,006	54,744
62	YDC107_5199	*finQ*	FinQ domain protein	56,533	56,159
63	YDC107_5200		conserved hypothetical protein	57,208	56,996
64	YDC107_5201		Hok/Gef family protein	56,851	57,006
65	YDC107_5202		conserved hypothetical protein	57,562	57,804
66	YDC107_5203		conserved hypothetical protein	58,045	57,869
67	YDC107_5204		HEAT-like repeat family protein	58,437	58,646
68	YDC107_5205		putative surface exclusion protein	59,380	58,718
69	YDC107_5206	*traY*	conjugal transfer/type IV secretion DotA/TraY family protein	61,619	59,451
70	YDC107_5207	*traX*	putative TraX-like protein	62,300	61,716
71	YDC107_5208	*traW*	putative TraW lipoprotein	63,531	62,329
72	YDC107_5209	*traV*	putative TraV protein	64,112	63,498
73	YDC107_5210		AAA-like domain protein	67,156	64,112
74	YDC107_5211	*traT*	putative incI1 conjugal transfer protein TraT	68,046	67,246
75	YDC107_5212	*traS*	putative incI1 conjugal transfer protein TraS	68,218	68,030
76	YDC107_5213	*traR*	putative TraR protein	68,686	68,282
77	YDC107_5214	*traQ*	putative TraQ protein	69,264	68,737
78	YDC107_5215	*traP*	putative TraP protein	69,968	69,264
79	YDC107_5216	*traO*	putative TraO protein	71,257	69,968
80	YDC107_5217	*traN*	putative TraN protein	72,243	71,260
81	YDC107_5218	*traM*	putative TraM protein	72,946	72,254
82	YDC107_5219	*traL*	putative TraL protein	73,290	72,943
83	YDC107_5220		putative DNA primase	77,075	73,308
84	YDC107_5221	*nuc*	endonuclease	77,716	77,165
85	YDC107_5222	*traK*	putative TraK protein	78,021	77,731
86	YDC107_5223	*traJ*	plasmid transfer ATPase TraJ	79,166	78,018
87	YDC107_5224	*traI*	putative TraI lipoprotein	79,981	79,163
88	YDC107_5225	*traH*	putative TraH protein	80,436	79,978
89	YDC107_5226	*traG*	putative TraG protein	81,412	80,831
90	YDC107_5227	*traF*	F plasmid transfer operon, TraF, family protein	82,677	81,475
91	YDC107_5228	*traE*	putative TraE protein	83,587	82,763
92	YDC107_5229		phage integrase family protein	84,892	83,738

Table S6. Annotation of plasmid pYDC107_70

CDS	Locus	Gene	Predicted Protein Function	Start	Stop
1	YDC107_5230	*repA*	initiator replication family protein, IncN	1	720
2	YDC107_5231		conserved hypothetical protein	765	2,255
3	YDC107_5232		conserved hypothetical protein	2,574	2,332
4	YDC107_5233		hypothetical protein	2,680	2,528
5	YDC107_5234		resolvase, N terminal domain protein	3,246	2,677
6	YDC107_5235	*intI1*	integron integrase family protein	4,652	3,639
7	YDC107_5236	*dfrA14*	dihydrofolate reductase type 1	4,808	5,281
8	YDC107_5237	*mobC*	bacterial mobilization family protein	5,502	5,768
9	YDC107_5238		hypothetical protein	5,768	5,878
10	YDC107_5239	*tnpA*	transposase IS66 family protein	5,911	6,675
11	YDC107_5240	*ecoRII*	type-2 restriction enzyme EcoRII	6,942	8,150
12	YDC107_5241	*dcm*	DNA (cytosine-5-)-methyltransferase family protein	9,617	8,184
13	YDC107_5242		conserved hypothetical protein	10,205	9,999
14	YDC107_5243		restriction endonuclease family protein	10,719	10,210
15	YDC107_5244		conserved hypothetical protein	11,218	10,907
16	YDC107_5245	*kikA*	putative KikA protein	11,568	11,254
17	YDC107_5246		conserved hypothetical protein	11,909	11,565
18	YDC107_5247	*korB*	H-NS histone family protein	12,230	11,925
19	YDC107_5248	*traL*	transglycosylase SLT domain protein	12,339	13,073
20	YDC107_5249	*korA*	putative KorA	13,082	13,363
21	YDC107_5250	*traM*	putative membrane protein	13,373	13,666
22	YDC107_5251	*traA*	type IV secretory pathway, VirB3-like family protein	13,716	14,033
23	YDC107_5252	*traB*	CagE, TrbE, VirB, component of type IV transporter system family protein	14,033	15,142
24	YDC107_5253	*arsR*	bacterial regulatory, ArsR family protein	15,981	15,784
25	YDC107_5254	*tnpA*	putative p12 protein	15,210	15,836
26	YDC107_5255		hypothetical protein	16,544	16,209
27	YDC107_5256	*nuc*	endonuclease	17,242	16,709
28	YDC107_5257	*traG*	type II/IV secretion system family protein	18,237	17,242
29	YDC107_5258	*traF*	TraF protein	19,439	18,279
30	YDC107_5259	*traO*	TraO protein	20,323	19,439
31	YDC107_5260	*traE*	VirB8 family protein	21,032	20,334
32	YDC107_5261	*traD*	TrbL/VirB6 plasmid conjugal transfer family protein	22,291	21,251
33	YDC107_5262	*eex*	putative entry exclusion protein, Eex	22,534	22,307
34	YDC107_5263	*traC*	TraC	23,255	22,542
35	YDC107_5264	*traB*	type IV secretion/conjugal transfer ATPase, VirB4 family protein	24,946	23,273
36	YDC107_5265		putative transposase	25,524	24,916
37	YDC107_5266		Tn3 transposase DDE domain protein	25,741	27,519
38	YDC107_5267		transposase family protein	27,598	28,602
39	YDC107_5268		integrase	28,960	28,784
40	YDC107_5269	*sul2*	dihydropteroate synthase	29,290	30,105
41	YDC107_5270	*aph(3'')-Ib*	Aminoglycoside 3'-phosphotransferase (EC 2.7.1.95) @ Streptomycin 3'-kinase StrA (EC 2.7.1.87)	30,166	30,969
42	YDC107_5271	*aph(6)-Id*	Aminoglycoside 3'-phosphotransferase 2 (EC 2.7.1.95) @ Streptomycin 3'-kinase StrB (EC 2.7.1.87)	31,176	31,805
43	YDC107_5272	bla_{TEM-1}	beta-lactamase TEM	33,386	32,526
44	YDC107_5273		resolvase, N terminal domain protein	33,820	33,569
45	YDC107_5274	bla_{OXA-9}	penicillin binding transpeptidase domain protein	34,910	34,086
46	YDC107_5275	*aadA*	nucleotidyltransferase domain protein	35,746	34,970
47	YDC107_5276	*aac(6')-Ib*	aminoglycoside N(6')-acetyltransferase type 1	36,433	35,828
48	YDC107_5277	*tnpR*	transposon Tn3 resolvase	37,173	36,616
49	YDC107_5278		transposase	37,337	38,245
50	YDC107_5279		phage integrase family protein	39,841	38,288
51	YDC107_5280		transposase	40,113	43,142
52	YDC107_5281		integrase core domain protein	43,249	44,274
53	YDC107_5282		PhoH-like family protein	44,271	45,050
54	YDC107_5283		conserved hypothetical protein	45,047	45,385
55	YDC107_5284	bla_{KPC-3}	carbapenem-hydrolyzing beta-lactamase KPC-3	45,437	46,318
56	YDC107_5285		transposase	47,887	46,568
57	YDC107_5286		Tn3 transposase DDE domain protein	48,338	50,353
58	YDC107_5287		putative transposase	50,406	51,032
59	YDC107_5288		Tn3 transposase DDE domain protein	51,068	51,496
60	YDC107_5289	*fipA*	putative FipA	51,871	51,506
61	YDC107_5290	*traI*	mobilization protein TraI	55,107	51,871
62	YDC107_5291	*traJ*	TraJ	56,636	55,107
63	YDC107_5292	*traK*	putative conjugal transfer protein, TraK	57,054	56,638
64	YDC107_5293	*stbA*	putative StbA	57,545	57,964
65	YDC107_5294	*stbB*	putative StbB	57,973	58,689
66	YDC107_5295	*stbC*	putative StbC protein	58,691	59,059
67	YDC107_5296	*orfD*	putative OrfD	59,241	59,585
68	YDC107_5297	*ccgAII*	putative CcgAII	60,016	59,696
69	YDC107_5298	*ccgAI*	putative CcgAI protein	60,250	60,071
70	YDC107_5299	*ardA*	antirestriction family protein	61,486	60,977
71	YDC107_5300		conserved hypothetical protein	62,511	62,254
72	YDC107_5301		hypothetical protein	62,529	62,819
73	YDC107_5302		conserved hypothetical protein	63,171	62,839
74	YDC107_5303		conserved hypothetical protein	63,302	63,631
75	YDC107_5304		conserved hypothetical protein	64,092	63,733
76	YDC107_5305	*ccgD*	putative CcgD protein	64,385	64,089
77	YDC107_5306		conserved hypothetical protein	65,043	64,498
78	YDC107_5307	*ccgEII*	putative CcgEII	65,510	65,271
79	YDC107_5308	*ardR*	putative antirestriction protein	65,873	65,520
80	YDC107_5309	*ardB*	antirestriction family protein	66,407	65,982
81	YDC107_5310	*mucA*	peptidase S24-like family protein	66,822	67,262
82	YDC107_5311	*mucB*	conserved hypothetical protein	67,250	68,515
83	YDC107_5312	*mpr*	SprT-like family protein	68,666	69,457
84	YDC107_5313	*ardK*	putative ArdK protein	69,813	69,472

Table S7. Annotation of plasmid pYDC107_41

CDS	Locus	Gene	Predicted Protein Function	Start	Stop
1	YDC107_5314	*trfA*	replication initiator A family protein	1	879
2	YDC107_5315	*klcA*	KlcA	2,031	2,498
3	YDC107_5316	*klcB*	putative protein KlcB	2,546	3,664
4	YDC107_5317		hypothetical protein	3,930	3,679
5	YDC107_5318	*kleE*	predicted KleE protein	4,023	4,346
6	YDC107_5319		conserved hypothetical protein	4,422	4,673
7	YDC107_5320		zeta toxin family protein	4,673	5,263
8	YDC107_5321	*trfB*	TrfB transcriptional repressor protein	5,465	5,809
9	YDC107_5322	*parA*	CobQ/CobB/MinD/ParA nucleotide binding domain protein	5,818	6,576
10	YDC107_5323	*korB*	transcriptional repressor protein KorB	6,576	7,589
11	YDC107_5324	*traO*	conjugal transfer protein TraO	8,170	8,523
12	YDC107_5325	*traN*	putative TraN	8,545	9,225
13	YDC107_5326	*traM*	protein TraM	9,823	9,383
14	YDC107_5327	*traL*	protein TraL	10,608	9,823
15	YDC107_5328	*traK*	putative TraK	11,042	10,608
16	YDC107_5329	*traJ*	protein TraJ	11,392	11,751
17	YDC107_5330	*traI*	protein TraI	11,780	14,413
18	YDC107_5331		type IV secretory system conjugative DNA transfer family protein	14,410	14,565
19	YDC107_5332	*traG*	conjugal transfer protein TraG	14,552	16,495
20	YDC107_5333	*traF*	conjugative transfer signal peptidase TraF	16,492	17,025
21	YDC107_5334		conserved hypothetical protein	17,045	17,374
22	YDC107_5335		hypothetical protein	17,371	17,697
23	YDC107_5336	*topB*	DNA topoisomerase III family protein	17,694	19,724
24	YDC107_5337	*traD*	putative TraD conjugal transfer protein	19,740	19,925
25	YDC107_5338	*traC*	DNA primase TraC	19,931	23,749
26	YDC107_5339		putative membrane protein	23,913	24,287
27	YDC107_5340		conserved hypothetical protein	25,361	24,375
28	YDC107_5341		hypothetical protein	25,642	25,436
29	YDC107_5342		putative membrane protein	25,932	26,129
30	YDC107_5343		hypothetical protein	27,102	26,218
31	YDC107_5344		putative membrane protein	27,332	27,697
32	YDC107_5345		resolvase, N terminal domain protein	27,781	28,395
33	YDC107_5346		helix-turn-helix transcriptional regulator	28,427	28,795
34	YDC107_5347		transglycosylase SLT domain, conjugal transfer protein	29,733	28,960
35	YDC107_5348		conserved hypothetical protein	30,057	29,749
36	YDC107_5349	*trbL*	P-type conjugative transfer protein TrbL	31,621	30,068
37	YDC107_5350	*trbJ*	P-type conjugative transfer protein TrbJ	32,615	31,833
38	YDC107_5351	*trbI*	bacterial conjugation TrbI-like family protein	34,022	32,628
39	YDC107_5352	*trbH*	conjugal transfer TrbH family protein	34,367	34,026
40	YDC107_5353	*trbG*	P-type conjugative transfer protein TrbG	35,362	34,472
41	YDC107_5354	*trbF*	VirB8 family protein	36,085	35,378
42	YDC107_5355	*trbE*	type IV secretion/conjugal transfer ATPase, VirB4 family protein	38,625	36,082
43	YDC107_5356	*trbD*	type IV secretory pathway, VirB3-like family protein	38,948	38,622
44	YDC107_5357	*trbC*	TrbC/VIRB2 family protein	39,334	38,951
45	YDC107_5358	*trbB*	P-type conjugative transfer ATPase TrbB	40,404	39,448
46	YDC107_5359	*trbA*	helix-turn-helix domain protein, TrbA	40,991	40,587
47	YDC107_5360	*ssb*	single-stranded DNA-binding family protein	41,096	41,470
